# The Impact of the First Trimester and Second Trimester Temperature, Air Pollutants, and Seasonal Variations on the Risk of Gestational Diabetes Mellitus in Twin Pregnancies

**DOI:** 10.1155/jdr/6633118

**Published:** 2025-09-23

**Authors:** Wei-Zhen Tang, Wei-Ze Xu, Yun-Ren Pan, Qin-Yu Cai, Li Wen, Hong-Yu Xu, Ying-Xiong Wang, Jia-Zheng Li, Tai-Hang Liu, Lan Wang

**Affiliations:** ^1^Department of Obstetrics and Gynecology, Women and Children's Hospital of Chongqing Medical University, Chongqing, China; ^2^Department of Bioinformatics, School of Basic Medical Sciences, Chongqing Medical University, Chongqing, China; ^3^The Joint International Research Laboratory of Reproduction and Development, Chongqing Medical University, Chongqing, China

**Keywords:** air pollutants, gestational diabetes mellitus, season, sensitive subtypes, temperature, twin pregnancy

## Abstract

**Background:** Recent studies have primarily focused on the impact of environmental factors on gestational diabetes mellitus (GDM) in singleton pregnancies, with limited research on their effects in twin pregnancies. This study investigates how seasonal variations and environmental exposures impact GDM incidence and its subtypes in twin pregnancies, a high-risk group.

**Methods:** In this retrospective analysis of 3769 twin pregnancies, we categorized recruited participants into GDM and non-GDM groups. We examined the effect of the screening season on oral glucose tolerance test (OGTT) glucose values and the incidence of GDM and its subtypes. Multivariable logistic regressions adjusted for confounders assessed the impact of first and second trimester temperatures and air pollutants on GDM risk. Interaction terms evaluated the combined effects of environmental factors on GDM incidence.

**Results:** Seasonal changes significantly influenced GDM risk, with summer presenting the highest risk (*p* < 0.05). The first trimester's cooler temperatures were inversely related to GDM; *T*_mean_ was significantly and negatively associated with 1-h PG and AUC for glucose, with adjusted *β* (95% CI) of −0.009 (−0.017, −0.001) and −0.719 (−1.406, −0.031), respectively. While warmer second trimester temperatures increased the risk, *T*_mean_ was positively associated with FBG, 1-h PG, 2-h PG, and AUC for glucose, with adjusted *β* (95% CI) of 0.003 (0.001, 0.005), 0.018 (0.009, 0.026), 0.019 (0.011, 0.027), and 1.723 (0.998, 2.448), respectively. Air pollutant exposure showed varying correlations with GDM risk, with ozone (O_3_) levels consistently posing a risk. Higher O_3_ exposure in the first and second trimesters was associated with increased odds of GDM, with OR (95% CI) of 1.057 (1.004, 1.112) and 1.052 (1.011, 1.096), respectively. Interaction analysis indicated that certain environmental conditions in the first trimester could reduce GDM risk, while others, particularly involving O_3_, increased it.

**Conclusion:** Environmental temperatures and air pollutants, especially O_3_, are associated with GDM risk in twin pregnancies, with differing effects between trimesters. These findings suggest that environmental factors should be considered in GDM screening and prevention strategies for twin pregnancies. Further research is needed to understand the underlying mechanisms and to develop trimester-specific interventions.

## 1. Introduction

Over the past two decades, the prevalence of GDM has been on the rise. According to the International Diabetes Federation, the global prevalence of GDM was approximately 14% in 2021 [1–3]. GDM is not only closely associated with adverse outcomes for both the mother and newborn, such as preeclampsia, cesarean delivery, preterm birth, macrosomia, and neonatal hypoglycemia [4], but it also increases the risk of developing Type 2 diabetes [5] and cardiovascular diseases later in life [6]. In twin pregnancies, the risk of GDM is further increased, and these populations are more susceptible to adverse obstetric outcomes such as pregnancy-induced hypertension, fetal growth restriction (FGR), and neonatal intensive care unit (NICU) admissions [7]. This heightened risk is partly due to the physiological differences between twin and singleton pregnancies, such as higher insulin resistance in twins [8, 9]. These differences may exacerbate the challenges in managing GDM in twin pregnancies. Therefore, understanding and controlling the risk factors for GDM in twin pregnancies is of significant importance for both clinical practice and research.

In recent years, increasing attention has been paid to the impact of environmental factors, such as seasonal variations, climate temperature, and air quality, on pregnancy-related diseases. Specifically, the influence of environmental changes on metabolic functions in pregnant women, particularly insulin sensitivity, may increase the risk of GDM, posing a challenge for the prevention of adverse pregnancy outcomes. Recent studies have focused on the effects of environmental factors such as seasonality, temperature fluctuations, and air pollutants on the incidence of GDM in singleton pregnancies, but the results have been inconsistent. Studies from Sweden, Australia, the United Kingdom, and Poland have all noted seasonal fluctuations in the diagnosis rate of GDM, with a peak during the summer [10–13]. However, research by Janghorbani et al. and Li et al. did not find seasonal variations in GDM [14, 15]. Moreover, evidence regarding the association between environmental temperature and GDM is inconsistent across different studies. Vasileiou et al. found no association between screening environmental temperature and GDM [16], while other studies indicate that higher environmental temperatures may be related to increased glucose levels during GDM screening tests [11, 16, 17]. Increasing evidence suggests that air pollutants in the air may increase the risk of GDM [18, 19]. Both animal and human studies indicate that exposure to air pollutants can impact glucose levels through various mechanisms, including insulin resistance [20], endothelial dysfunction [21, 22], and inflammatory responses [23, 24]. Given the high incidence of GDM in twin pregnancies and its impact on the health of pregnant women, identifying environmental risk factors for GDM is crucial for developing effective prevention measures [25]. Therefore, determining the environmental risk factors that impact GDM in twin pregnancies is essential for the development of prevention strategies [26].

Based on laboratory examinations, OGTT results can be categorized as normal glucose tolerance (NGT), isolated fasting hyperglycemia (GDM-IFH), isolated postloading hyperglycemia (GDM-IPH), or combined hyperglycemia (GDM-CH) [27]. Recent studies have found that different subtypes of GDM may represent distinct metabolic entities. Previous research has shown that GDM-IFH is closely related to hepatic insulin sensitivity and subsequent hepatic glucose production, whereas GDM-IPH is closely associated with muscle insulin resistance [28, 29]. Past studies have also indicated that GDM-IFH is closely associated with adverse pregnancy outcomes and benefits less from dietary and lifestyle therapy, thus more likely requiring insulin treatment [30, 31]. Given that environmental factors may have different effects on different GDM subtypes in twin pregnancies, it is thus of significant importance to systematically elucidate the impact of environmental exposures on GDM in twin pregnancies.

This study is aimed at exploring the potential sustained impact of screening season, environmental temperature, and air pollutants during the first trimester and the second trimester on glucose levels at various time points during the OGTT and the incidence of GDM and its subtypes in twin pregnancies. The findings will provide an important basis for preventive strategies in twin pregnancies, helping to mitigate the adverse effects of environmental factors on the health of this special population.

## 2. Materials and Methods

### 2.1. Ethics Statement

This study was approved by the ethics committee of Women's and Children's Hospital of Chongqing Medical University (ID: 2022-011-01).

### 2.2. Data Availability Statement

To safeguard patient privacy, all personally identifiable information was removed from the cases, and all acquired data were kept anonymous but are available from the corresponding authors on reasonable request.

### 2.3. Ethics Approval and Consent to Participate

This study is in accordance with the Declaration of Helsinki. All methods were carried out in accordance with the relevant guidelines and regulations. The participants' legal guardian or next of kin provided written informed consent for this study.

### 2.4. Data Collection

All relevant clinical data came from the electronic medical record (EMR) database of Women's and Children's Hospital of Chongqing Medical University, including basic information about pregnant women and pregnancy-related information.

### 2.5. Assessment of Ambient Temperature and Air Pollutant Exposure

**Figure 1:**
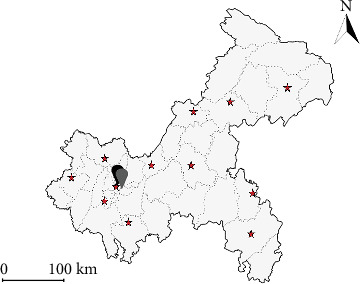
Geographical map and distribution of meteorological stations in Chongqing.

Chongqing is a city located in the southwestern part of China, with a total area of 82,400 km^2^, approximately 75% of which is mountainous. As of the end of 2021, Chongqing has 38 districts and counties with a total population of 31.20 million. The city is primarily divided into four main regions: central, western, northeastern, and southeastern (Figure 1). The central region features relatively flat terrain, advanced economic development, and a higher rate of urbanization. As the industrial base of Southwest China, industry plays a critical role in economic development, and industrial pollution is a key factor contributing to environmental contamination.

Chongqing hosts 12 primary meteorological stations located in Fengjie, Wanzhou, Qianjiang, Youyang, Liangping, Changshou, Fengdu, Hechuan, Shapingba, Jiangjin, Qijiang, and Dazu (Figure 1). From December 2017 to December 2022, daily maximum (*T*_max_), minimum (*T*_min_), and mean (*T*_mean_) temperature records in degrees Celsius were obtained from the Chongqing Meteorological Service. The daily *T*_mean_ was calculated based on 24-h temperature recordings. The diurnal temperature range (DTR) was defined by calculating the difference between the daily *T*_max_ and *T*_min_. Environmental pollution data, including air pollutants (PM_2.5_), inhalable particulate matter (PM_10_), sulfur dioxide (SO_2_), nitrogen dioxide (NO_2_), carbon monoxide (CO), and ozone (O_3_), were collected from the China Meteorological Data Sharing Service System covering the period from January 2017 to December 2022. Due to privacy concerns, individual home addresses of participants were not available. Consequently, exposure estimates were derived based on the geocoding of hospital locations, which may not accurately reflect the actual exposure levels experienced by pregnant women. This approach could introduce exposure misclassification, as it assumes that environmental conditions at the hospital locations are representative of those at participants' residences [32, 33]. Seasonal variables were categorized as follows: winter (December to February), spring (March to May), summer (June to August), and autumn (September to November) [11, 34].

### 2.6. Study Design

**Figure 2:**
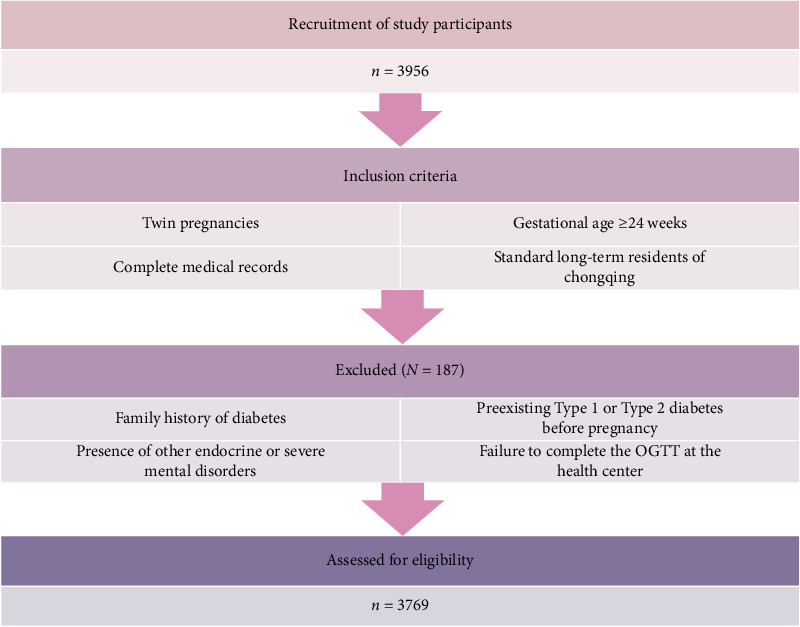
Flowchart of this retrospective cohort study.

The inclusion criteria for this retrospective cohort study were pregnant women who had their first prenatal care visit at the Women and Children's Hospital of Chongqing Medical University between January 2017 and December 2022, standard long-term residents of Chongqing, twin pregnancies, complete medical records, and gestational age ≥ 24 weeks. The exclusion criteria were pre-existing Type 1 or Type 2 diabetes before pregnancy, family members with diabetes, severe mental disorders, other endocrine diseases (in addition to diabetes and other endocrine disorders, including thyroid, adrenal, and hypothalamic diseases), and failure to complete the oral glucose tolerance test at a health center. The operational definition of the criterion of “long-term residency in Chongqing with no plans to move during pregnancy” was as follows: residence within a 30-min travel time from the delivery hospital. This definition was verified through geospatial analysis of participants' self-reported communities against real-time traffic data by the study team. This process ensured residential stability during pregnancy. This analysis was conducted at the Women's and Children's Hospital of Chongqing Medical University (Figure 2).

### 2.7. Definition

According to the diagnostic criteria established by the International Association of Diabetes and Pregnancy Study Groups (IADPSG) [35], pregnant women undergo an OGTT between 24 and 28 weeks of gestation, with glucose measurement conducted using the glucose oxidase method (Hitachi 7600-110 Automatic Biochemical Analyzer, produced by Hitachi, Tokyo, Japan). The expectant mothers are required to fast for 8–12 h overnight, and fasting venous blood is collected the following morning to measure blood glucose levels. Afterward, they ingest 75 g of glucose, and venous blood is collected again 1 and 2 h postglucose ingestion.

The area under the curve (AUC) for glucose refers to the total AUC plotted against time during the OGTT, which reflects the overall elevation of blood glucose levels over a certain period. The calculation is typically performed using the trapezoidal rule, which involves dividing the AUC into multiple trapezoids, summing their areas, or by using more precise mathematical integration methods to determine this area, thereby quantifying the overall glucose response.

The diagnostic criteria for GDM are as follows: fasting blood glucose ≥ 5.1 mmol/L (92 mg/dL), 1-h postglucose blood glucose ≥ 10.0 mmol/L (180 mg/dL), or 2-h postglucose blood glucose ≥ 8.5 mmol/L (153 mg/dL). A diagnosis of GDM can be made if any one of these criteria is met. Based on the results of the OGTT, if a pregnant woman's fasting blood glucose is ≥ 5.1 mmol/L but her 1 and 2-h postglucose blood glucose levels are within normal ranges, she is classified as having GDM-IFH; if her 1 and 2-h postglucose blood glucose levels are ≥ 10.0 and ≥ 8.5 mmol/L, respectively, she is classified as having GDM-IPH. Women exceeding both fasting and postprandial glucose thresholds are considered to have GDM-CH [27].

### 2.8. Statistical Analysis

To describe the characteristics of the participants, continuous data were summarized as means with standard deviations for normally distributed data and medians with interquartile ranges (IQRs) for skewed distributions. Categorical data were presented as counts and percentages. The Mann–Whitney *U* test was used to compare age, prepregnancy body mass index (BMI), and blood glucose values at different times during the OGTT, while the chi-square test was used for count data in the presence or absence of GDM groups. For variables with chi-square *p* values close to 0.05, Fisher's exact test was employed. The influence of different seasons on blood glucose values at various times during the OGTT (including FBG, 1-h PG, and 2-h PG) and AUC glucose was compared using the Mann–Whitney *U* test. The chi-square test was also used to compare the incidence of overall GDM and its subtypes (GDM-IFH, GDM-IPH, and GDM-CH). Furthermore, this study conducted multivariate linear regression and multivariate logistic regression analyses, adjusting for potential confounders such as age, BMI, primigravid status, family history of hyperglycemia, and season of pregnancy, to explore the impact of screening season and environmental temperature and air pollutant levels in the first trimester and the second trimester on blood glucose values at different OGTT times and the risk of GDM occurrence. Dual-pollutant models were conducted to test the joint effects of simultaneously fitted air pollutants. Pollutants with correlation coefficients higher than 0.9 were not fitted together. Finally, we investigated the interaction effects of four temperature indicators (*T*_mean_, *T*_min_, *T*_max_, and DTR) and six air pollutants (PM_2.5_, PM_10_, SO_2_, NO_2_, CO, and O_3_) during the first trimester and the second trimester on the risk of GDM in twin pregnancies by adding multiplicative interaction terms in the generalized linear model of logistic regression. Adjusted odds ratios (aORs) with 95% CI and *p* values for interaction adjusted for confounding factors were calculated. Interaction effects were visualized using the “visreg” package in R. All statistical analyses were performed using SPSS Software Version 26.0 and R Version 4.4.2. This comprehensive analytical strategy, employing a variety of statistical methods, aids in our understanding of the impact of seasonality, environmental temperatures, and air pollutants during the first trimester and the second trimester on the risk of GDM in twin pregnancies, as well as the complex interaction effects that may exist between these factors.

## 3. Result

### 3.1. Baseline Characteristics and Environmental Factors During the First and Second Trimesters in Twin Pregnancies

In this study, we included 3769 pregnant women with twin pregnancies, among whom 1063 were diagnosed with GDM, representing 28.2% of the cohort. As shown in Table 1, women with GDM exhibited higher average age and BMI compared to those without GDM (*p* < 0.001). They also had a significantly greater prevalence of family history of hyperglycemia and multiparity (*p* < 0.001). Furthermore, women with GDM were more likely to conceive during the winter months. Other potential influencing factors, such as family history of hypertension and chorionicity, did not show significant differences between the groups. Further analysis of the relationship between environmental temperature variables (*T*_mean_, *T*_max_, *T*_min_, DTR) and air pollutant levels (PM_2.5_, PM_10_, SO_2_, NO_2_, CO, O_3_) during the first and second trimesters of pregnancy revealed that, regardless of early or the second trimester, temperature variables were significantly negatively correlated with PM_2.5_, PM_10_, SO_2_, NO_2_, and CO and significantly positively correlated with O_3_ (*p* < 0.001) (Table S1 and Table S2). In addition, Spearman correlation analysis of the six pollutants revealed that O_3_ was negatively correlated with the other pollutants throughout the study period (Table S3).

### 3.2. Association of Screening Season With OGTT Glucose Levels and GDM Subtype Incidence in Twin Pregnancies

To investigate the influence of environmental factors on the risk of GDM in twin pregnancies, an initial comparative analysis of blood glucose levels at different time points during the OGTT and the incidence of GDM and its subtypes during various screening seasons was conducted. The study found that the season of screening significantly impacted blood glucose levels at all time points of the OGTT and the AUC for glucose (*p* < 0.01). Although the season of screening did not significantly impact the overall incidence of GDM in twin pregnancies, it had a significant impact on the incidence of GDM-IFH and GDM-IPH subtypes. The highest risk for GDM-IFH occurred during winter screenings, while the highest risk for GDM-IPH was during summer screenings, which could partly explain why there was no significant difference in the incidence rates of total GDM and GDM-CH (Table S4). Multifactorial logistic regression, adjusted for confounders, revealed that compared to winter, the overall risk of GDM for pregnant women screened in summer increased by 33.4% (aOR = 1.334, *p* = 0.007). For the subtypes of GDM, the risks of GDM-IPH in spring and summer compared to winter increased by 39.5% and 107.2%, respectively (*p* < 0.01). Conversely, the risks of GDM-IFH in spring and summer were significantly lower than in winter (*p* < 0.05) (Table S2).

### 3.3. Association of Environmental Temperature With OGTT Glucose Values and Incidence of GDM Subtypes in Twin Pregnancies

To delve deeper into the impact of persistent environmental factors at different stages of pregnancy on the incidence of GDM and its subtypes in twin pregnancies, multivariate models were used to analyze the effects of environmental temperatures and air pollutants during the first and second trimesters on glucose levels at various OGTT time points, as well as on the incidence of GDM and its subtypes. Regarding environmental temperature, during the first trimester, the DTR had no significant effect on glucose levels at any OGTT time points. However, *T*_max_ was negatively correlated with 1-h PG, while *T*_mean_ and *T*_min_ were not only negatively correlated with 1-h glucose levels but also with the AUC for glucose. In contrast, during the second trimester, *T*_mean_, *T*_max_, *T*_min_, and DTR were all positively correlated with glucose values at all OGTT time points (*p* < 0.05). When analyzing the impact of climatic temperature on the risk of GDM and its subtypes, the first trimester *T*_mean_, *T*_max_, and *T*_min_ were negatively correlated with the overall risk of GDM (*p* < 0.05), with no significant effect found on other GDM subtypes. In the second trimester, increases in *T*_mean_, *T*_max_, and *T*_min_ were significantly associated with an increased risk of GDM-IPH, leading to a rise in the overall risk of GDM. Additionally, the impact of DTR exhibited a stage-specific change, from no significant effect on GDM and its subtypes in the first trimester to a significant increase in the risk of overall GDM, GDM-IPH, and GDM-CH during the second trimester.

### 3.4. Association of Air Pollutant Exposure With OGTT Glucose Values and Incidence of GDM Subtypes in Twin Pregnancies

In terms of air pollutants, for glucose values at various OGTT time points, during the first trimester, an increase in CO concentration was significantly negatively correlated with 2-h PG and the AUC for glucose (2-h PG, aOR = −1.110, *p* < 0.05; AUC for glucose, aOR = −87.169, *p* < 0.05). Conversely, elevated O_3_ concentrations were significantly positively correlated with FBG, 2-h PG, and AUC glucose (FBG, aOR = 0.014, *p* < 0.05; 2-h PG, aOR = 0.047, *p* < 0.05; AUC for glucose, aOR = 4.104, *p* < 0.05). Moving into the second trimester, the impact of O_3_ extended to 1-h PG (1-h PG, aOR = 0.060, *p* < 0.05), showing a significant positive correlation, while other pollutants besides O_3_ were significantly negatively correlated with glucose values at all OGTT time points. When assessing the influence of air pollutants on the risk of GDM and its subtypes, the results indicated that the first trimester CO concentration was negatively correlated with GDM-IPH (aOR = 0.194, *p* < 0.05), but this association was no longer significant in the second trimester. In contrast, increased O_3_ concentration significantly elevated the risk of overall GDM and GDM-IPH, in the first and second trimesters (*p* < 0.05). The effects of other air pollutants on GDM and its subtypes were not significant (Table 2). To further assess the robustness of these associations, we conducted effect estimations using two-pollutant models. Detailed results are presented in Table S5. After adjusting for copollutants, the significance of some pollutants' effects on blood glucose indicators and GDM was reduced, but the overall trends remained consistent (Table S5).

### 3.5. Interactive Effects of Environmental Temperature and Air Pollutant Exposure on the Incidence of GDM in Twin Pregnancies

**Figure 3:**
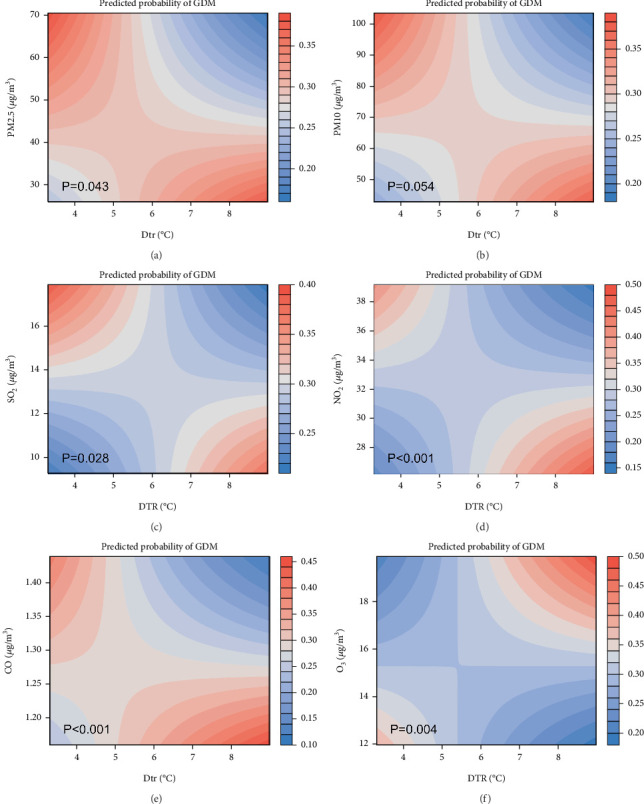
(a–f) Interaction effects of various air pollutants and DTR during the first trimester on the occurrence of GDM. Interaction effects based on prepregnancy BMI, age, in vitro fertilization (IVF), scarred uterus, gravidity, and primiparity. The analysis was conducted using a generalized linear model with interaction effects. The color scale on the right side of the contour plot represents the occurrence rate of GDM from the lower to the upper limit. Abbreviations: DTR: diurnal temperature range; BMI: body mass index; PM_2.5_: fine particulate matter; PM_10_: inhalable particulate matter; SO_2_: sulfur dioxide; NO_2_: nitrogen dioxide; CO: carbon monoxide; O_3_: ozone.

Finally, this study conducted a comprehensive analysis of the interaction effects between temperature indices and concentrations of air pollutants during the first and second trimesters. In the first trimester, significant interaction effects were observed. High PM_2.5_ concentrations with a large DTR and low PM_2.5_ concentrations with a small DTR were associated with a reduced risk of GDM (Figures S1, S2, S3, S4, S5, S6, and S7). Conversely, combinations of high PM_2.5_ with a small DTR and low PM_2.5_ with a large DTR increased the risk (PM_2.5_: *p* for interaction = 0.043) (Figure 3), while the trend for O_3_ was the opposite (O_3_: *p* for interaction = 0.004) (Figure 3), which is consistent with previous findings (Table S2). Additionally, combinations of high CO concentrations with higher *T*_max_ were shown to interactively reduce the risk of GDM, whereas combinations of low CO with lower *T*_max_ were associated with increased GDM risk (CO: *p* for interaction = 0.029) (Figure S1). Notably, these interaction effects underwent a significant shift during the second trimester. Positive interaction effects observed in the first trimester mostly transitioned to negative in the second trimester. Particularly, the interaction effect between O_3_ and DTR in the second trimester displayed a completely opposite trend compared to the first trimester: combinations of high O_3_ with a large DTR and low O_3_ with a small DTR both showed interaction effects that reduced the risk of GDM in the second trimester, whereas combinations of high O_3_ with a small DTR and low O_3_ with a large DTR were associated with increased GDM risk (O_3_: *p* for interaction = 0.026) (Figure S4). Aside from these explicit interactions, no significant interactive effects were observed among other combinations of temperature indices and concentrations of air pollutants (Table S6).

## 4. Discussion

This study has effectively demonstrated that environmental factors, including screening season, temperature, and air pollutants, significantly impact the risk of GDM in twin pregnancies, aligning with our initial hypothesis. Our findings reveal that summer screenings are associated with the highest GDM risk, while cooler temperatures in the first trimester correlate with lower GDM risk. In contrast, warmer temperatures during the second trimester elevate this risk. Additionally, we found that exposure to air pollutants, particularly O_3_, is linked to increased GDM risk, highlighting the complex interactions between environmental conditions and glucose metabolism. These results indicate that integrating environmental factors into GDM screening and prevention strategies for twin pregnancies is crucial for protecting the health of this vulnerable population.

Previous studies have consistently shown that the risk of screening for GDM is significantly higher in summer compared to winter, which is in agreement with our findings [36]. However, our study further reveals that the distribution of GDM subtypes also exhibits a seasonal dependency pattern: the risk for GDM-IFH is highest when screened in winter, while the risk for GDM-IPH peaks during summer screenings [13]. This seasonal relationship persists even after adjusting for confounding factors. The different incidence trends for distinct subtypes may reflect varied pathophysiological mechanisms. The elevated OGTT glucose values and higher incidence of GDM-IPH in summer could be related to physiological adaptations to temperature changes. Previous research has suggested that apparent glucose tolerance (measured by OGTT values) could be impacted by fluctuations in environmental temperature, potentially due to a rise in core body temperature leading to a redistribution of blood flow from the skin to the visceral vascular bed, resulting in reduced glucose uptake by peripheral tissues and increased OGTT glucose values [37]. Studies indicate that vitamin D deficiency may impact glucose metabolism [38]. The increase in fasting blood glucose levels in winter could be associated with low serum vitamin D levels, which are more common in cold climates. Additionally, the rise in fasting glucose may also be linked to the seasonal rhythms of melatonin, as melatonin can directly inhibit insulin secretion [38]. Our study also suggests that seasonal changes can cause fasting blood glucose and postload glucose values to exhibit inverse trends. This is consistent with the findings of Shen et al., who discovered a significant positive correlation between fasting glucose, glycated hemoglobin (HBA1C), and insulin resistance index (HOMA-IR) with the winter season. Conversely, elevated postprandial glucose levels were observed in summer [17]. These seasonal variations in blood glucose levels partially explain the seasonal differences in the incidence of GDM subtypes. Moreover, the influence of seasonal factors on the risk of GDM in twin pregnancies may be related to seasonal changes in circadian rhythms. Seasonal variations in physical activity and dietary patterns, as well as opportunities for exposure to green spaces and sunlight, may also impact the seasonal variations in GDM and glucose outcomes [39–41].

Building on these insights, most current research attributes the seasonal variation of GDM primarily to the influence of environmental temperatures. Therefore, this study delves further into the impact of these temperatures during the first and second trimesters on the risk of GDM in twin pregnancies. The study finds that during the second trimester, increases in *T*_mean_, *T*_max_, and *T*_min_ are not only positively correlated with glucose values at various times but are also significantly associated with an increased risk of GDM-IPH, and these temperature rises likewise lead to an increased overall risk of GDM. In contrast, during the first trimester, *T*_mean_, *T*_max_, and *T*_min_ show a negative correlation with the overall incidence of GDM, with no significant association with the occurrence of other GDM subtypes. These results suggest that higher temperatures in the second trimester are a significant factor in the increased risk of GDM, consistent with conclusions from a study on singleton pregnancies conducted in Guangzhou, China, which found that the same temperature variables had the strongest effect during the second trimester [32]. Additionally, this study observes that an increase in the DTR goes from having no significant impact on GDM and its subtypes in the first trimester to significantly increasing the risk of overall GDM, GDM-IPH, and GDM-CH during the second trimester. Research by Stuebe et al. also indicated that the sensitive period for DTR occurs in the second trimester, which may reflect a reduced capacity for pregnant women to adapt to temperature changes [42]. Temperature variations may impact maternal blood circulation, leading to a redistribution of blood between the arterial and venous systems, causing an increase in glucose levels in the veins [43, 44]. This increase in glucose levels could exacerbate insulin resistance typically occurring in the second trimester [45]. Several physiological mechanisms may elucidate how temperature influences glucose metabolism and insulin resistance, particularly during crucial periods of gestational development [37, 43, 46]. One fundamental factor is *β*-cell dysfunction, which can result from thermal stress. Elevated temperatures increase oxidative stress on pancreatic *β*-cells, impairing their ability to secrete insulin in response to high blood glucose levels [47, 48]. Additionally, insulin resistance, a key feature of GDM, can be affected by environmental temperatures [37, 43, 46]. Higher ambient temperatures are associated with alterations in blood viscosity, leading to hemoconcentration, where the blood has a higher concentration of cells and reduced plasma volume. This condition may cause temporary hyperglycemia, as the circulating insulin may be insufficient to maintain normal glucose homeostasis. Furthermore, increased temperatures can cause arteriolization, disrupting blood flow dynamics and dilating peripheral blood vessels. This dilation may affect the distribution and utilization of glucose in tissues, impairing insulin signaling pathways and exacerbating insulin resistance [37, 43, 46]. Recent research highlights the role of adipose tissue inflammation and thermoregulation. Elevated temperatures trigger inflammatory cytokines like TNF-*α* and IL-6 in adipose tissue, which disrupt insulin signaling and contribute to insulin resistance [49, 50]. Additionally, thermal stress may impair the thermogenic activity of brown adipose tissue (BAT), reducing energy expenditure and promoting fat accumulation, further exacerbating glucose intolerance during pregnancy [51, 52]. Currently, there is a relative paucity of research on the impact of environmental temperatures on GDM subtypes in twin pregnancies, especially studies comparing them with singleton pregnancies. Future research should consider the effects of environmental temperatures on GDM subtypes and the differences between singleton and twin pregnancies. This exploration will contribute to a more comprehensive understanding of how variations in environmental temperatures influence the risk of developing GDM. Ultimately, such insights could lead to more targeted recommendations for the prevention and management of GDM, enhancing maternal and fetal health outcomes.

In parallel, studies examining the association between air pollutants, particularly PM_2.5_, and GDM have sparked considerable debate. Although early observational studies have identified a potential link between PM_2.5_ and GDM, the findings have been inconsistent. In this study, we observed that an increase in PM_2.5_ concentrations during the second trimester was significantly inversely correlated with the overall risk of GDM in twin pregnancies and the occurrence of GDM-IPH, aligning with results from a study in California [53]. However, other studies have indicated that exposure to PM_2.5_ during the second trimester is associated with a higher incidence of GDM [54]. Moreover, a cohort study in Massachusetts did not find a significant association between PM_2.5_ and GDM [55]. Thus, further research is required to observe the effects of PM_2.5_ exposure during different gestational periods and to analyze GDM and its various subtypes. Our results also suggest that, compared to PM_2.5_, PM_10_ and SO_2_ have a lesser impact on GDM, which is consistent with previous meta-analysis findings [56]. Although PM_10_ and SO_2_ were significantly inversely correlated with 1-h PG, 2-h PG, and AUC glucose and PM_10_ had a significant risk-reducing effect on GDM-IPH, they did not significantly impact the overall incidence of GDM. However, studies by Zeng et al. found that PM_10_ is associated with an increased risk of GDM-IPH [23], and research by Cao et al. indicated that exposure to PM_10_ prepregnancy and during pregnancy significantly increased the risk of GDM [57]. These discrepancies may be related to differences in exposure levels, windows, analytical methods, and effect modifiers used in the studies, as well as the unique physiological characteristics of twin pregnancies. The study also found that PM_10_ significantly increased the risk of GDM-IFH, suggesting that the second trimester might be a sensitive period for the impact of air pollutant exposure on GDM-IFH risk. Additionally, we found that increases in O_3_ levels from early to the second trimester were not only associated with elevated blood glucose levels but also significantly heightened the risk of overall GDM and GDM-IPH. These findings align with prior research, which indicates that O_3_ exposure can trigger pulmonary inflammatory responses [58]. Such inflammatory responses are known to be linked with insulin resistance and hyperglycemia. Furthermore, long-term exposure to O_3_ has been shown to elevate oxidative stress and inflammation in adipose tissue, thereby exacerbating insulin resistance [59]. Given that insulin resistance is a critical factor in the development of GDM [60, 61], it stands to reason that exposure to O_3_ could increase the risk of GDM, particularly in twin pregnancies, by further promoting this insulin resistance. Recent studies also suggest that air pollution can influence epigenetic mechanisms during pregnancy, further complicating the development of GDM. For instance, exposure to pollutants such as PM_2.5_ and O_3_ has been linked to altered DNA methylation patterns in maternal and fetal tissues, which may affect gene expression related to inflammation and metabolic regulation [62, 63]. These epigenetic changes could exacerbate insulin resistance by altering the expression of key genes involved in adipogenesis and insulin signaling [64]. Furthermore, prenatal exposure to pollutants has been shown to affect the development of the placental epigenome, potentially modifying the maternal–fetal nutrient exchange and contributing to the long-term risk of metabolic disorders like GDM [65–67].

Furthermore, while the individual effects of temperature and air pollutants on GDM have been explored separately, few studies have addressed the complex interaction effect between temperature and air pollutants. In a study assessing the impact of extreme temperatures on GDM in singleton pregnancies, no interaction effect was found between temperature and air pollutants [32]. However, in our study, we discovered varying multiplicative interaction effects between temperature and air pollutants at different times. This discrepancy may be due to the different interaction effect models applied and the periods studied. Additionally, our study elucidated the interaction effects between pollutants and temperature at different gestational stages. In the first trimester, a complex interaction effect was observed between multiple air pollutants (PM_2.5_, SO_2_, NO_2_, and CO) and the DTR on the risk of GDM in twin pregnancies, which seems to correspond with the Spearman correlation analysis results. As DTR increases, the concentrations of PM_2.5_, SO_2_, NO_2_, and CO tend to decrease, potentially resulting in fewer GDM cases concurrently exposed to high DTR and high concentrations of PM_2.5_, SO_2_, NO_2_, and CO, ultimately reducing the risk of GDM in twin pregnancies under these severe conditions. In the second trimester, these interaction effects were not observed, and further research is needed to substantiate this finding. Interestingly, an interaction effect between O_3_ and DTR was observed, altering the risk of GDM from the first trimester to the second trimester in twin pregnancies. An increase in DTR may induce thermal inversions [68]. A cohort study from Wuhan, China, involving over 30,000 pregnant women, indicated that thermal inversions and O_3_ exposure during the first trimester and the second trimester have a synergistic additive interaction effect in increasing the risk of GDM [69]. However, in our study, this effect was present only in the first trimester and shifted during the second trimester, potentially because the baseline level of O_3_ exposure was higher in the second trimester. The body may have an adaptive dose–response relationship to O_3_ [70], whereby high concentrations of O_3_ in the second trimester could trigger more complex endocrine regulatory mechanisms, activating defense systems such as antioxidant enzymes, which could offset the negative impact of DTR to some extent. Furthermore, few studies have shown that CO exposure is associated with an increased risk of GDM. A study from Guangzhou, China, involving approximately 40,000 individuals, revealed that the first trimester CO exposure was associated with an increased risk of GDM but did not explore the related interaction effect [71]. The complex interaction effect between CO and temperature requires further investigation. Future research should explore the intricate pathological processes in twin pregnancies with GDM under exposure to air pollutants and temperature.

## 5. Limitations

This study is a single-center retrospective analysis where all baseline information was obtained through the electronic record access system of the medical center. Some covariate data were missing from the records, including chronic diseases and/or medication, employment, marital status, and living alone or with others. Considering that pregnant women spend a significant amount of time indoors (whether at home or in the hospital) and often use air conditioning, there may be substantial differences between indoor and outdoor temperatures that were not fully considered in this study, which could impact the risk of GDM. Secondly, due to the lack of precise residential location data for the pregnant women, we were unable to accurately match individual pollution exposure levels using the inverse distance weighting (IDW) method. This limitation may have introduced some misclassification of air pollution exposure among the participants. While previous studies have suggested that there is no significant difference between temperature and pollution data estimated using the IDW method and those from nearby meteorological stations, we acknowledge that this method could still result in some exposure misclassification, particularly in areas where exposure data may not accurately reflect the actual residential environment [32, 33]. As such, we recognize the potential bias in exposure estimation and encourage future studies to incorporate more precise residential data or consider alternative methods, such as clustering around hospitals, to minimize exposure misclassification and improve the accuracy of environmental exposure assessments. Thirdly, the results of the two-pollutant models indicated that the significance of some air pollutants' effects on GDM in twin pregnancies was attenuated. However, the overall trends remained consistent with the main findings. The attenuation observed in some pollutant effects can be attributed to the potential confounding effects of co-occurring pollutants. While high correlations between pollutants limit the direct fitting of all pollutants together, we used dual-pollutant models to address this issue. Techniques such as elastic net regression or principal component analysis (PCA) could be considered in future studies to further mitigate the influence of collinearity and to refine our understanding of the joint effects of air pollutants. Finally, we did not fit dual-pollutant models due to the strong correlation among most air pollutants in the air. Potential confounding effects of coexisting air pollutants could be possible in our effect estimates. In addition to these six types of air pollutant, other pollutants might also impact the risk of GDM in twin pregnancies. Despite these limitations, our study provides valuable insights into the potential impact of environmental factors on the risk of GDM in twin pregnancies. Future research could focus on the cumulative impact of multiple climate-related factors (such as humidity, precipitation, urban green space, noise, and the interaction effect of other environmental factors) on GDM and related outcomes in twin pregnancies.

## Figures and Tables

**Table 1:** Characteristics of GDM and non-GDM participants included in this study.

**Characteristics**	**GDM (** **n** = 1063**)**	**Non-GDM (** **n** = 2706**)**	**p** ** value**
Age, M (Q_1_, Q_3_)	32.00 (29.00, 34.00)	31.00 (28.00, 33.00)	< 0.001⁣^∗^
BMI, M (Q_1_, Q_3_)	22.00 (20.16, 24.04)	21.08 (19.47, 23.05)	< 0.001⁣^∗^
Primigravida, *n* (%)	451 (42.43)	1323 (48.89)	< 0.001⁣^∗^
Nulliparity, *n* (%)	856 (80.53)	2234 (82.56)	0.144
Family history of hypertension, *n* (%)	159 (14.96)	353 (13.05)	0.123
Family history of hyperglycemia, *n* (%)	121 (11.38)	117 (4.32)	< 0.001⁣^∗^
Chorionic, *n* (%)			0.086
DCDA	884 (83.16)	2185 (80.75)	
Non-DCDA	179 (16.84)	521 (19.25)	
FBG, M (Q_1_, Q_3_)	4.80 (4.40, 5.20)	4.40 (4.20, 4.60)	< 0.001⁣^∗^
1-h PG, M (Q_1_, Q_3_)	10.10 (9.20, 11.00)	7.80 (6.80, 8.70)	< 0.001⁣^∗^
2-h PG, M (Q_1_, Q_3_)	8.70 (7.80, 9.60)	6.50 (5.80, 7.30)	< 0.001⁣^∗^
Season of conception (*n*, %)			0.024⁣^∗^
Spring (March–May)	531 (19.62)	247 (23.24)	
Summer (June–August)	708 (26.16)	258 (24.27)	
Fall (September–November)	696 (25.72)	240 (22.58)	
Winter (December–February)	771 (28.49)	318 (29.92)	

Abbreviations: 1-h PG, 1-h plasma glucose; 2-h PG, 2-h plasma glucose; BMI, body mass index; DCDA, dichorionic diamniotic.

⁣^∗^p < 0.05.

**Table 2:** The impact of temperature and air pollutants on OGTT glucose values, GDM, and its subtypes during different periods of pregnancy.

	**a*β* (95% CI)**				**aOR (95% CI)**			
	**FBG**	**1-h PG**	**2-hPG**	**AUC for glucose**	**GDM**	**GDM-IFH**	**GDM-IPH**	**GDM-CH**
First trimester							
*T*_mean_	−0.001 (−0.003, 0.001)	−0.009 (−0.017, −0.001)	−0.005 (−0.012, 0.003)	−0.719 (−1.406, −0.031)	0.987 (0.978, 0.998)	1.002 (0.980, 1.024)	0.989 (0.977, 1.001)	0.988 (0.970, 1.006)
*T*_max_	−0.000 (−0.002, 0.002)	−0.007 (−0.015, −0.000)	−0.003 (−0.010, 0.003)	−0.557 (−1.170, 0.055)	0.990 (0.981, 0.999)	1.002 (0.983, 1.023)	0.991 (0.980, 1.001)	0.990 (0.974, 1.006)
*T*_min_	−0.001 (−0.003, 0.001)	−0.011 (−0.020, −0.002)	−0.006 (−0.014, 0.002)	−0.884 (−1.648, −0.120)	0.985 (0.974, 0.996)	1.001 (0.976, 1.026)	0.987 (0.974, 1.000)	0.985 (0.965, 1.005)
DTR	0.005 (−0.003, 0.013)	−0.004 (−0.035, 0.026)	0.009 (−0.019, 0.037)	0.175 (−2.430, 2.781)	0.991 (0.954, 1.030)	1.035 (0.950, 1.126)	0.983 (0.940, 1.029)	0.997 (0.931, 1.068)
PM_2.5_	0.000 (−0.001, 0.001)	0.002 (−0.003, 0.008)	−0.000 (−0.005, 0.004)	0.128 (−0.335, 0.591)	1.003 (0.996, 1.010)	1.003 (0.988, 1.018)	0.999 (0.991, 1.008)	1.007 (0.995, 1.019)
PM_10_	0.000 (−0.001, 0.001)	0.002 (−0.002, 0.006)	−0.000 (−0.004, 0.004)	0.117 (−0.224, 0.459)	1.003 (0.998, 1.008)	1.002 (0.991, 1.013)	1.000 (0.994, 1.006)	1.006 (0.997, 1.015)
SO_2_	0.004 (−0.004, 0.011)	0.013 (−0.016, 0.041)	0.003 (−0.024, 0.029)	0.961 (−1.494, 3.417)	1.024 (0.988, 1.062)	1.036 (0.958, 1.122)	0.998 (0.956, 1.041)	1.048 (0.983, 1.118)
NO_2_	−0.002 (−0.007, 0.004)	−0.007 (−0.028, 0.014)	−0.013 (−0.032, 0.007)	−0.834 (−2.661, 0.993)	0.995 (0.969, 1.022)	1.019 (0.961, 1.082)	0.983 (0.953, 1.015)	1.001 (0.954, 1.050)
CO	−0.133 (−0.359, 0.093)	−0.831 (−1.668, 0.005)	−1.110 (−1.875, −0.345)	−87.169 (−158.91, −15.428)	0.369 (0.128, 1.065)	0.907 (0.089, 9.292)	0.194 (0.055, 0.684)	1.368 (0.207, 9.046)
O_3_	0.014 (0.003, 0.025)	0.038 (−0.003, 0.079)	0.047 (0.010, 0.085)	4.104 (0.613, 7.595)	1.057 (1.004, 1.112)	1.009 (0.902, 1.130)	1.067 (1.004, 1.133)	1.051 (0.960, 1.151)
Second trimester							
*T*_mean_	0.003 (0.001, 0.005)	0.018 (0.009, 0.026)	0.019 (0.011, 0.027)	1.723 (0.998, 2.448)	1.024 (1.013, 1.035)	1.012 (0.988, 1.036)	1.024 (1.011, 1.037)	1.016 (0.996, 1.035)
*T*_max_	0.003 (0.001, 0.005)	0.016 (0.009, 0.024)	0.017 (0.010, 0.024)	1.584 (0.936, 2.232)	1.022 (1.012, 1.032)	1.011 (0.989, 1.032)	1.022 (1.010, 1.033)	1.015 (0.998, 1.033)
*T*_min_	0.003 (0.001, 0.006)	0.019 (0.009, 0.028)	0.020 (0.012, 0.029)	1.831 (1.029, 2.630)	1.025 (1.013, 1.037)	1.013 (0.987, 1.040)	1.025 (1.011, 1.039)	1.016 (0.995, 1.038)
DTR	0.010 (0.002, 0.019)	0.073 (0.042, 0.105)	0.067 (0.038, 0.095)	6.704 (4.005, 9.404)	1.105 (1.061, 1.151)	1.032 (0.944, 1.127)	1.099 (1.047, 1.153)	1.089 (1.013, 1.172)
PM_2.5_	−0.001 (−0.003, 0.000)	−0.012 (−0.017, −0.007)	−0.012 (−0.017, −0.007)	−1.108 (−1.566, −0.651)	0.988 (0.981, 0.995)	1.003 (0.988, 1.018)	0.985 (0.977, 0.994)	0.992 (0.980, 1.004)
PM_10_	−0.001 (−0.002, 0.00)	−0.009 (−0.013, −0.005)	−0.009 (−0.012, −0.005)	−0.813 (−1.153, −0.473)	0.991 (0.986, 0.996)	1.002 (0.991, 1.013)	0.994 (0.985, 1.003)	0.994 (0.985, 1.003)
SO_2_	−0.005 (−0.012, 0.003)	−0.051 (−0.079, −0.024)	−0.052 (−0.077, −0.027)	−4.780 (−7.145, −2.415)	0.954 (0.921, 0.988)	1.017 (0.942, 1.098)	0.949 (0.911, 1.001)	0.956 (0.897, 1.018)
NO_2_	−0.001 (−0.006, 0.004)	−0.033 (−0.053, −0.014)	−0.032 (−0.049, −0.014)	−2.992 (−4.643, −1.341)	0.971 (0.948, 0.995)	1.018 (0.965, 1.074)	0.960 (0.933, 0.988)	0.990 (0.948, 1.034)
CO	−0.151 (−0.367, 0.065)	−1.223 (−2.022, −0.423)	−0.971 (−1.702, −0.239)	−107.011 (−175.564, −38.457)	0.321 (0.117, 1.085)	1.387 (0.151, 12.749)	0.310 (0.093, 1.031)	0.328 (0.053, 2.037)
O_3_	−0.002 (−0.011, 0.007)	0.060 (0.028, 0.092)	0.050 (0.021, 0.080)	5.043 (2.265, 7.820)	1.052 (1.011, 1.096)	0.955 (0.870, 1.049)	1.093 (1.043, 1.145)	0.973 (0.903, 1.048)

*Note:* a*β* and aOR values were adjusted for age, BMI, primigravida, family history of hyperglycemia, and season of conception.

Abbreviations: 1-h PG, 1-h plasma glucose; 2-h PG, 2-h plasma glucose; AUC for glucose, area under the curve for glucose; CO, carbon monoxide; DTR, diurnal temperature range; FBG, fasting blood glucose; GDM, gestational diabetes mellitus; GDM-CH, gestational diabetes mellitus–combined hyperglycemia; GDM-IFH, gestational diabetes mellitus–impaired fasting hyperglycemia; GDM-IPH, gestational diabetes mellitus–impaired postprandial hyperglycemia; NO_2_, nitrogen dioxide; O_3_, ozone; PM_2.5_, fine particulate matter; PM_10_, inhalable particulate matter; SO_2_, sulfur dioxide; *T*_max_, maximum temperature; *T*_mean_, mean temperature; *T*_min_, minimum temperature.

## Data Availability

The data underlying this article will be provided by the corresponding authors on reasonable request.

## Supporting Information

Supporting Information Additional supporting information can be found online in the Supporting Information section. The supporting information presents the effects of interactions between various air pollutants and mean, maximum, and minimum temperatures (*T*_mean_, *T*_max_, and *T*_min_) on the incidence of gestational diabetes mellitus (GDM) in twin pregnancies during the first and second trimesters. Figure S1. Interaction effects of various air pollutants and *T*_max_ during the first trimester on the occurrence of GDM. (A) PM_2.5_ and *T*_max_; (B) PM_10_ and *T*_max_; (C) SO_2_ and *T*_max_; (D) NO_2_ and *T*_max_; (E) CO and *T*_max_; (F) O_3_ and *T*_max_. Interaction effects based on prepregnancy BMI, age, in vitro fertilization, scarred uterus, gravidity, and primiparity. The analysis was conducted using a generalized linear model with interaction effects. The color scale on the right side of the contour plot represents the occurrence rate of GDM from the lower to the upper limit. Abbreviations: *T*_max_: maximum temperature; BMI: body mass index; PM_2.5_: fine particulate matter; PM_10_: inhalable particulate matter; SO_2_: sulfur dioxide; NO_2_: nitrogen dioxide; CO: carbon monoxide; O_3_: ozone. Figure S2. Interaction effects of various air pollutants and *T*_mean_ during the first trimester on the occurrence of GDM. (A) PM_2.5_ and *T*_mean_; (B) PM_10_ and *T*_mean_; (C) SO_2_ and *T*_mean_; (D) NO_2_ and *T*_mean_; (E) CO and *T*_mean_; (F) O_3_ and *T*_mean_. Interaction effects based on prepregnancy BMI, age, in vitro fertilization, scarred uterus, gravidity, and primiparity. The analysis was conducted using a generalized linear model with interaction effects. The color scale on the right side of the contour plot represents the occurrence rate of GDM from the lower to the upper limit. Abbreviations: *T*_mean_: mean temperature; BMI: body mass index; PM_2.5_: fine particulate matter; PM_10_: inhalable particulate matter; SO_2_: sulfur dioxide; NO_2_: nitrogen dioxide; CO: carbon monoxide; O_3_: ozone. Figure S3. Interaction effects of various air pollutants and *T*_min_ during the first trimester on the occurrence of GDM. (A) PM_2.5_ and *T*_min_; (B) PM_10_ and *T*_min_; (C) SO_2_ and *T*_min_; (D) NO_2_ and *T*_min_; (E) CO and *T*_min_; (F) O_3_ and *T*_min_. Interaction effects based on prepregnancy BMI, age, in vitro fertilization, scarred uterus, gravidity, and primiparity. The analysis was conducted using a generalized linear model with interaction effects. The color scale on the right side of the contour plot represents the occurrence rate of GDM from the lower to the upper limit. Abbreviations: *T*_min_: minimum temperature; BMI: body mass index; PM_2.5_: fine particulate matter; PM_10_: inhalable particulate matter; SO_2_: sulfur dioxide; NO_2_: nitrogen dioxide; CO: carbon monoxide; O_3_: ozone. Figure S4. Interaction effects of various air pollutants and DTR during the second trimester on the occurrence of GDM. (A) PM_2.5_ and DTR; (B) PM_10_ and DTR; (C) SO_2_ and DTR; (D) NO_2_ and DTR; (E) CO and DTR; (F) O_3_ and DTR. Interaction effects based on prepregnancy BMI, age, in vitro fertilization, scarred uterus, gravidity, and primiparity. The analysis was conducted using a generalized linear model with interaction effects. The color scale on the right side of the contour plot represents the occurrence rate of GDM from the lower to the upper limit. Abbreviations: DTR: diurnal temperature range; BMI: body mass index; PM_2.5_: fine particulate matter; PM_10_: inhalable particulate matter; SO_2_: sulfur dioxide; NO_2_: nitrogen dioxide; CO: carbon monoxide; O_3_: ozone. Figure S5. Interaction effects of various air pollutants and *T*_max_ during the second trimester on the occurrence of GDM. (A) PM_2.5_ and *T*_max_; (B) PM_10_ and *T*_max_; (C) SO_2_ and *T*_max_; (D) NO_2_ and *T*_max_; (E) CO and *T*_max_; (F) O_3_ and *T*_max_. Interaction effects based on prepregnancy BMI, age, in vitro fertilization, scarred uterus, gravidity, and primiparity. The analysis was conducted using a generalized linear model with interaction effects. The color scale on the right side of the contour plot represents the occurrence rate of GDM from the lower to the upper limit. Abbreviations: *T*_max_: maximum temperature; BMI: body mass index; PM_2.5_: fine particulate matter; PM_10_: inhalable particulate matter; SO_2_: sulfur dioxide; NO_2_: nitrogen dioxide; CO: carbon monoxide; O_3_: ozone. Figure S6. Interaction effects of various air pollutants and *T*_mean_ during the second trimester on the occurrence of GDM. (A) PM_2.5_ and *T*_mean_; (B) PM_10_ and *T*_mean_; (C) SO_2_ and *T*_mean_; (D) NO_2_ and *T*_mean_; (E) CO and *T*_mean_; (F) O_3_ and *T*_mean_. Interaction effects based on prepregnancy BMI, age, in vitro fertilization, scarred uterus, gravidity, and primiparity. The analysis was conducted using a generalized linear model with interaction effects. The color scale on the right side of the contour plot represents the occurrence rate of GDM from the lower to the upper limit. Abbreviations: *T*_mean_: mean temperature; BMI: body mass index; PM_2.5_: fine particulate matter; PM_10_: inhalable particulate matter; SO_2_: sulfur dioxide; NO_2_: nitrogen dioxide; CO: carbon monoxide; O_3_: ozone. Figure S7. Interaction effects of various air pollutants and *T*_min_ during the second trimester on the occurrence of GDM. (A) PM_2.5_ and *T*_min_; (B) PM_10_ and *T*_min_; (C) SO_2_ and *T*_min_; (D) NO_2_ and *T*_min_; (E) CO and *T*_min_; (F) O_3_ and *T*_min_. Interaction effects based on prepregnancy BMI, age, in vitro fertilization, scarred uterus, gravidity, and primiparity. The analysis was conducted using a generalized linear model with interaction effects. The color scale on the right side of the contour plot represents the occurrence rate of GDM from the lower to the upper limit. Abbreviations: *T*_min_: minimum temperature; BMI: body mass index; PM_2.5_: fine particulate matter; PM_10_: inhalable particulate matter; SO_2_: sulfur dioxide; NO_2_: nitrogen dioxide; CO: carbon monoxide; O_3_: ozone. Table S1. The average values of environmental temperature and air pollutants during first trimester to second trimester. Abbreviations: *T*_mean_: mean temperature; *T*_max_: maximum temperature; *T*_min_: minimum temperature; DTR: diurnal temperature range; PM_2.5_: fine particulate matter; PM_10_: inhalable particulate matter; SO_2_: sulfur dioxide; NO_2_: nitrogen dioxide; CO: carbon monoxide; O_3_: ozone. Table S2. Analysis of the correlation between environmental temperature and air pollutants during first trimester to second trimester. Abbreviations: *T*_mean_: mean temperature; *T*_max_: maximum temperature; *T*_min_: minimum temperature; PM_2.5_: fine particulate matter; PM_10_: inhalable particulate matter; SO_2_: sulfur dioxide; NO_2_: nitrogen dioxide; CO: carbon monoxide; O_3_: ozone. Table S3. Spearman analysis results regarding the relationship between six air pollutants. Abbreviations: PM_2.5_: fine particulate matter; PM_10_: inhalable particulate matter; SO_2_: sulfur dioxide; NO_2_: nitrogen dioxide; CO: carbon monoxide; O_3_: ozone. Table S4. The influence of the screening season on GDM and its subtypes. Abbreviations: GDM: gestational diabetes mellitus; GDM-IFH: gestational diabetes mellitus–impaired fasting hyperglycemia; GDM-IPH: gestational diabetes mellitus–impaired postprandial hyperglycemia; GDM-CH: gestational diabetes mellitus–combined hyperglycemia; BMI: body mass index. Table S5. In the dual-pollutant model, the impact of temperature and air pollutants on OGTT glucose values, GDM, and its subtypes during different periods of pregnancy. The aOR values were adjusted for age, BMI, whether the woman is primigravida, family history of hyperglycemia, and the season of pregnancy. Abbreviations: FBG: fasting blood glucose; 1-h PG: 1-h plasma glucose; 2-h PG: 2-h plasma glucose; AUC for glucose: area under the curve for glucose; GDM: gestational diabetes mellitus; GDM-IFH: gestational diabetes mellitus–impaired fasting hyperglycemia; GDM-IPH: gestational diabetes mellitus–impaired postprandial hyperglycemia; GDM-CH: gestational diabetes mellitus–combined hyperglycemia; PM_2.5_: fine particulate matter; PM_10_: inhalable particulate matter; SO_2_: sulfur dioxide; NO_2_: nitrogen dioxide; CO: carbon monoxide; O_3_: ozone; BMI: body mass index. Table S6. The interaction effect between temperature and air pollutants on the incidence of GDM during different periods of pregnancy. Abbreviations: GDM: gestational diabetes mellitus; GDM-IFH: gestational diabetes mellitus–impaired fasting hyperglycemia; GDM-IPH: gestational diabetes mellitus–impaired postprandial hyperglycemia; GDM-CH: gestational diabetes mellitus–combined hyperglycemia; BMI: body mass index. Abbreviations: *T*_mean_: mean temperature; *T*_max_: maximum temperature; *T*_min_: minimum temperature; DTR: diurnal temperature range; PM_2.5_: fine particulate matter; PM_10_: inhalable particulate matter; SO_2_: sulfur dioxide; NO_2_: nitrogen dioxide; CO: carbon monoxide; O_3_: ozone; BMI: body mass index.
