# Supplementary material for: The Impact of the First Trimester and Second Trimester Temperature, Air Pollutants, and Seasonal Variations on the Risk of Gestational Diabetes Mellitus in Twin Pregnancies
Source: J Diabetes Res. 2025 Sep 23;2025:6633118. doi: 10.1155/jdr/6633118 (PMC12483729; doi:10.1155/jdr/6633118)
Supplement: Supporting Information — Additional supporting information can be found online in the Supporting Information section. The supporting information presents the effects of interactions between various air pollutants and mean, maximum, and minimum temperatures (Tmean, Tmax, and Tmin) on the incidence of gestational diabetes mellitus (GDM) in twin pregnancies during the first and second trimesters. Figure S1. Interaction effects of various air pollutants and Tmax during the first trimester on the occurrence of GDM. (A) PM2.5 and Tmax; (B) PM10 and Tmax; (C) SO2 and Tmax; (D) NO2 and Tmax; (E) CO and Tmax; (F) O3 and Tmax. Interaction effects based on prepregnancy BMI, age, in vitro fertilization, scarred uterus, gravidity, and primiparity. The analysis was conducted using a generalized linear model with interaction effects. The color scale on the right side of the contour plot represents the occurrence rate of GDM from the lower to the upper limit. Abbreviations: Tmax: maximum temperature; BMI: body mass index; PM2.5: fine particulate matter; PM10: inhalable particulate matter; SO2: sulfur dioxide; NO2: nitrogen dioxide; CO: carbon monoxide; O3: ozone. Figure S2. Interaction effects of various air pollutants and Tmean during the first trimester on the occurrence of GDM. (A) PM2.5 and Tmean; (B) PM10 and Tmean; (C) SO2 and Tmean; (D) NO2 and Tmean; (E) CO and Tmean; (F) O3 and Tmean. Interaction effects based on prepregnancy BMI, age, in vitro fertilization, scarred uterus, gravidity, and primiparity. The analysis was conducted using a generalized linear model with interaction effects. The color scale on the right side of the contour plot represents the occurrence rate of GDM from the lower to the upper limit. Abbreviations: Tmean: mean temperature; BMI: body mass index; PM2.5: fine particulate matter; PM10: inhalable particulate matter; SO2: sulfur dioxide; NO2: nitrogen dioxide; CO: carbon monoxide; O3: ozone. Figure S3. Interaction effects of various air pollutants and Tmin during the first tr [file 6633118.f1.docx]

**The Impact of the first trimester and the second trimester Temperature, Air Pollutants, and Seasonal Variations on the Risk of Gestational Diabetes Mellitus in Twin Pregnancies**

Wei-Zhen Tang^a,b^, Wei-Ze Xu^a,b^, Yun-Ren Pan^a,b^, Qin-Yu Cai^a,b^, Li Wen^a^, Hong-Yu Xu^a,b^, Ying-Xiong Wang^b,c^, Jia-Zheng Li^a,b^, Tai-Hang Liu^b,c,^*, Lan Wang^a,^*

**Affiliations:**

^a^ Department of Obstetrics and Gynecology, Women and Children’s Hospital of Chongqing Medical University, Chongqing, 401147, China.

^b^ Department of Bioinformatics, School of Basic Medical Sciences, Chongqing Medical University, Chongqing, 400016, China.

^c^ The Joint International Research Laboratory of Reproduction and Development, Chongqing Medical University, Chongqing, 400016, China.

***Correspondence:**

*Tai-Hang Liu (E-mail Address: [liuth@cqmu.edu.cn);](mailto:liuth@cqmu.edu.cn);) Box 197, Chongqing Medical University, No.1 Yixueyuan Rd, Chongqing, 400016, PR China. Tel.: +86 023 68485868.

*Lan Wang (E-mail Address: [wanglan120@outlook.com)](mailto:wanglan120@outlook.com)); Women and Children's Hospital of Chongqing Medical University, No. 120 Longshan Rd, Yubei District, Chongqing, 401147, China. Tel: +86 23 63840868

**Running title**: Environmental Impact on GDM in Twins


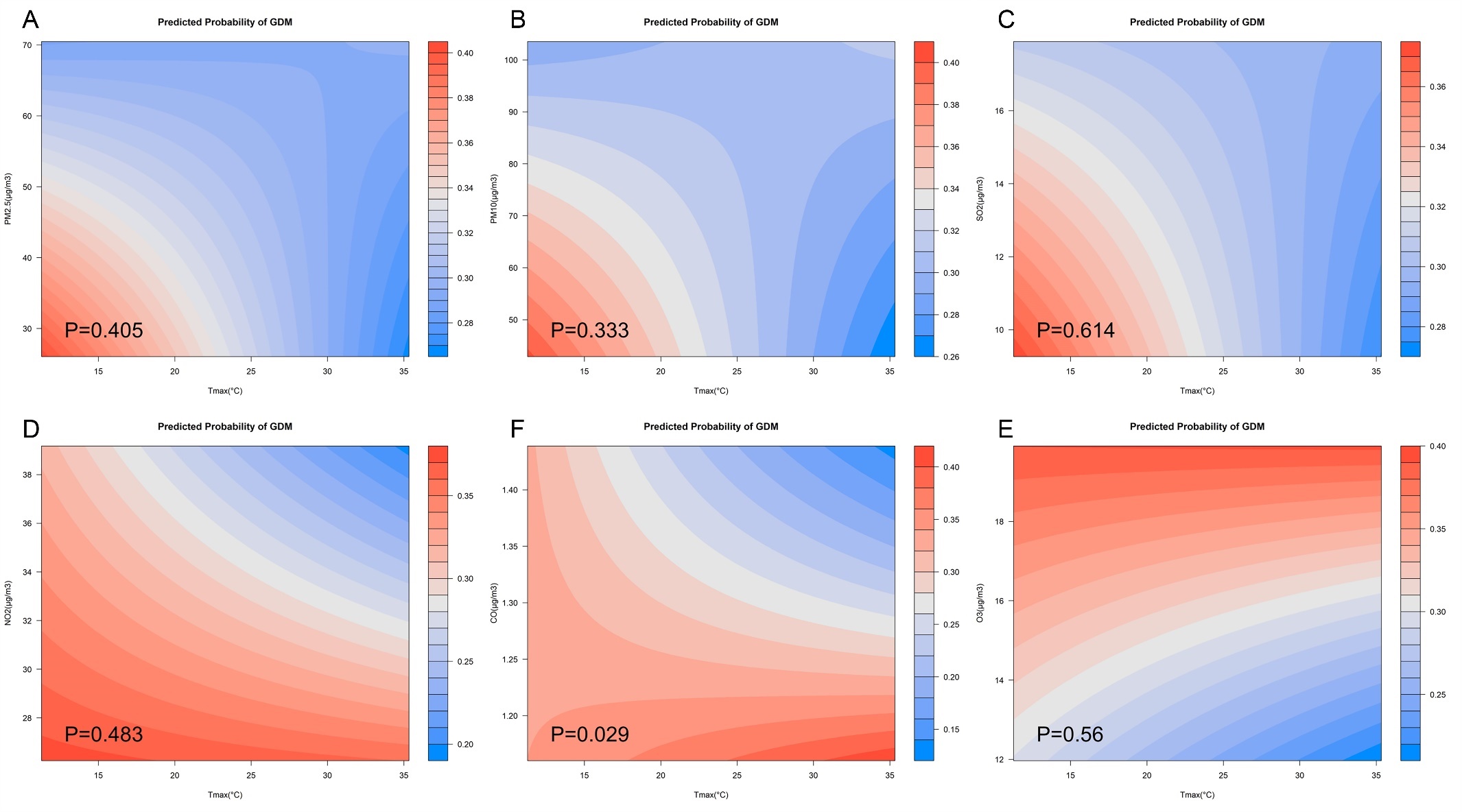


Supplementary Figure 1. Interaction effects of various air pollutants and T_max_ during the 1st trimester on the occurrence of GDM. (A) PM_2.5_ and T_max_; (B) PM_10_ and T_max_; (C) SO_2_ and T_max_; (D) NO_2_ and T_max_; (E) CO and T_max_; (F) O_3_ and T_max_. Interaction effects based on pre-pregnancy BMI, age, in vitro fertilization, scarred uterus, gravidity, and primiparity. The analysis was conducted using a generalized linear model with interaction effects. The color scale on the right side of the contour plot represents the occurrence rate of GDM from the lower to the upper limit.

Abbreviation:T_max_: Maximum Temperature; BMI: Body mass index; PM_2.5_: Fine particulate matter; PM_10_: Inhalable particulate matter; SO_2:_ Sulfur dioxide; NO_2_: Nitrogen dioxide; CO: Carbon monoxide; O_3_: Ozone.


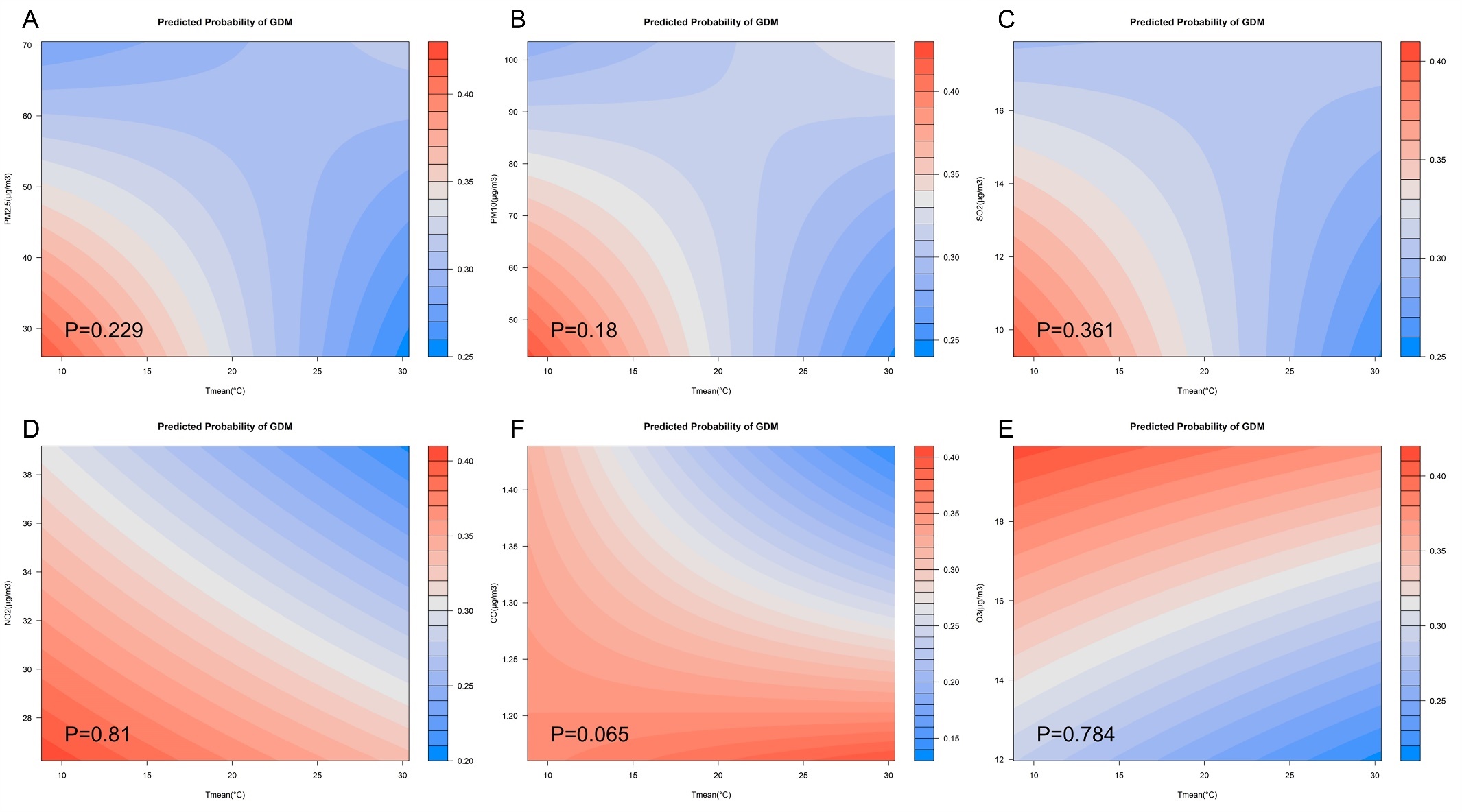


Supplementary Figure 2. Interaction effects of various air pollutants and Tmean during the 1st trimester on the occurrence of GDM. (A) PM_2.5_ and T_mean_; (B) PM_10_ and T_mean_; (C) SO_2_ and T_mean_; (D) NO_2_ and T_mean_; (E) CO and T_mean_; (F) O_3_ and T_mean_. Interaction effects based on pre-pregnancy BMI, age, in vitro fertilization, scarred uterus, gravidity, and primiparity. The analysis was conducted using a generalized linear model with interaction effects. The color scale on the right side of the contour plot represents the occurrence rate of GDM from the lower to the upper limit.

Abbreviation: T_mean_: Mean Temperature; BMI: Body mass index; PM_2.5_: Fine particulate matter; PM_10_: Inhalable particulate matter; SO_2:_ Sulfur dioxide; NO_2_: Nitrogen dioxide; CO: Carbon monoxide; O_3_: Ozone.


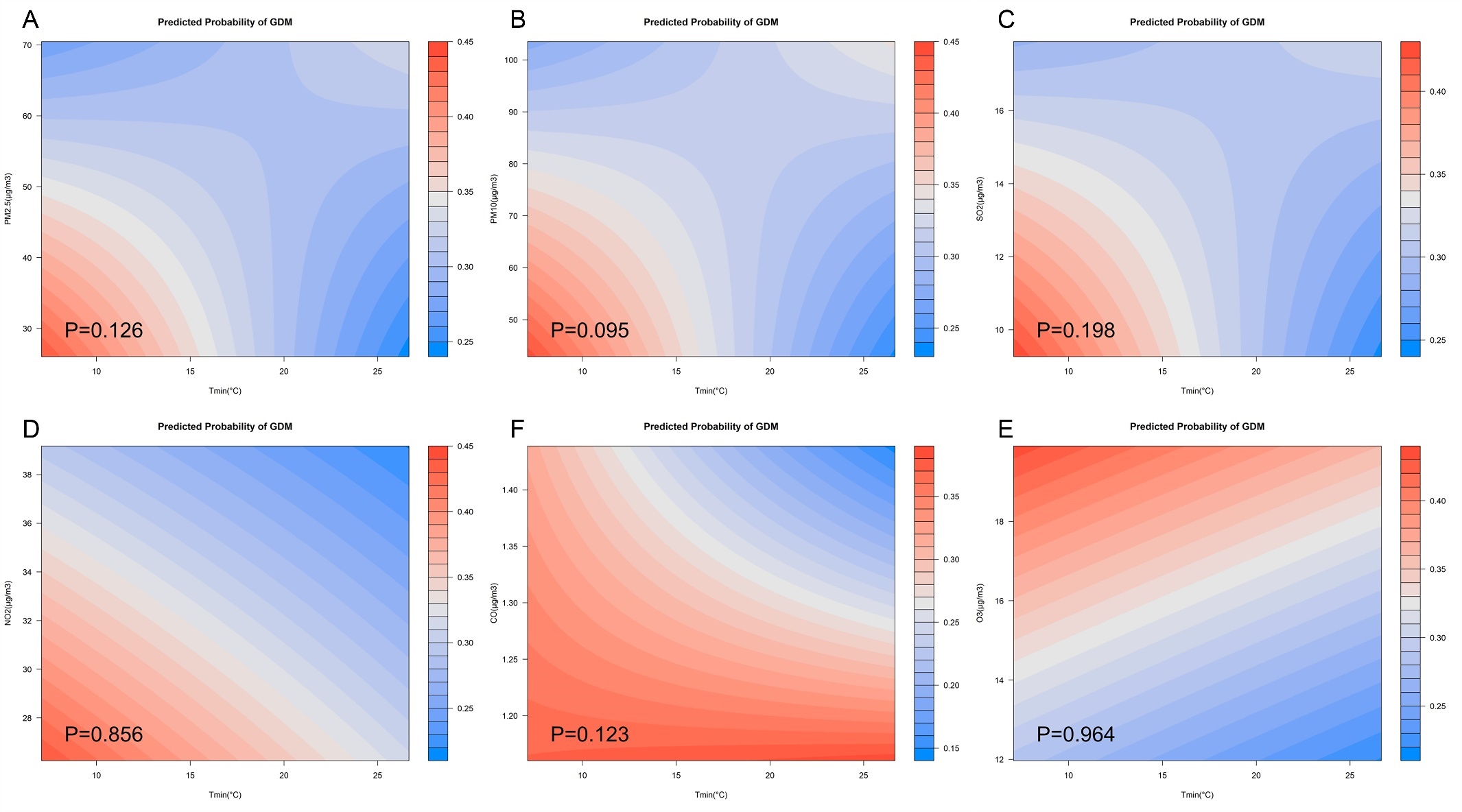


Supplementary Figure 3. Interaction effects of various air pollutants and Tmin during the 1st trimester on the occurrence of GDM. (A) PM_2.5_ and T_min_; (B) PM_10_ and T_min_; (C) SO_2_ and T_min_; (D) NO_2_ and T_min_; (E) CO and T_min_; (F) O_3_ and T_min_. Interaction effects based on pre-pregnancy BMI, age, in vitro fertilization, scarred uterus, gravidity, and primiparity. The analysis was conducted using a generalized linear model with interaction effects. The color scale on the right side of the contour plot represents the occurrence rate of GDM from the lower to the upper limit.

Abbreviation: T_min_: Minimum Temperature; BMI: Body mass index; PM_2.5_: Fine particulate matter; PM_10_: Inhalable particulate matter; SO_2:_ Sulfur dioxide; NO_2_: Nitrogen dioxide; CO: Carbon monoxide; O_3_: Ozone.


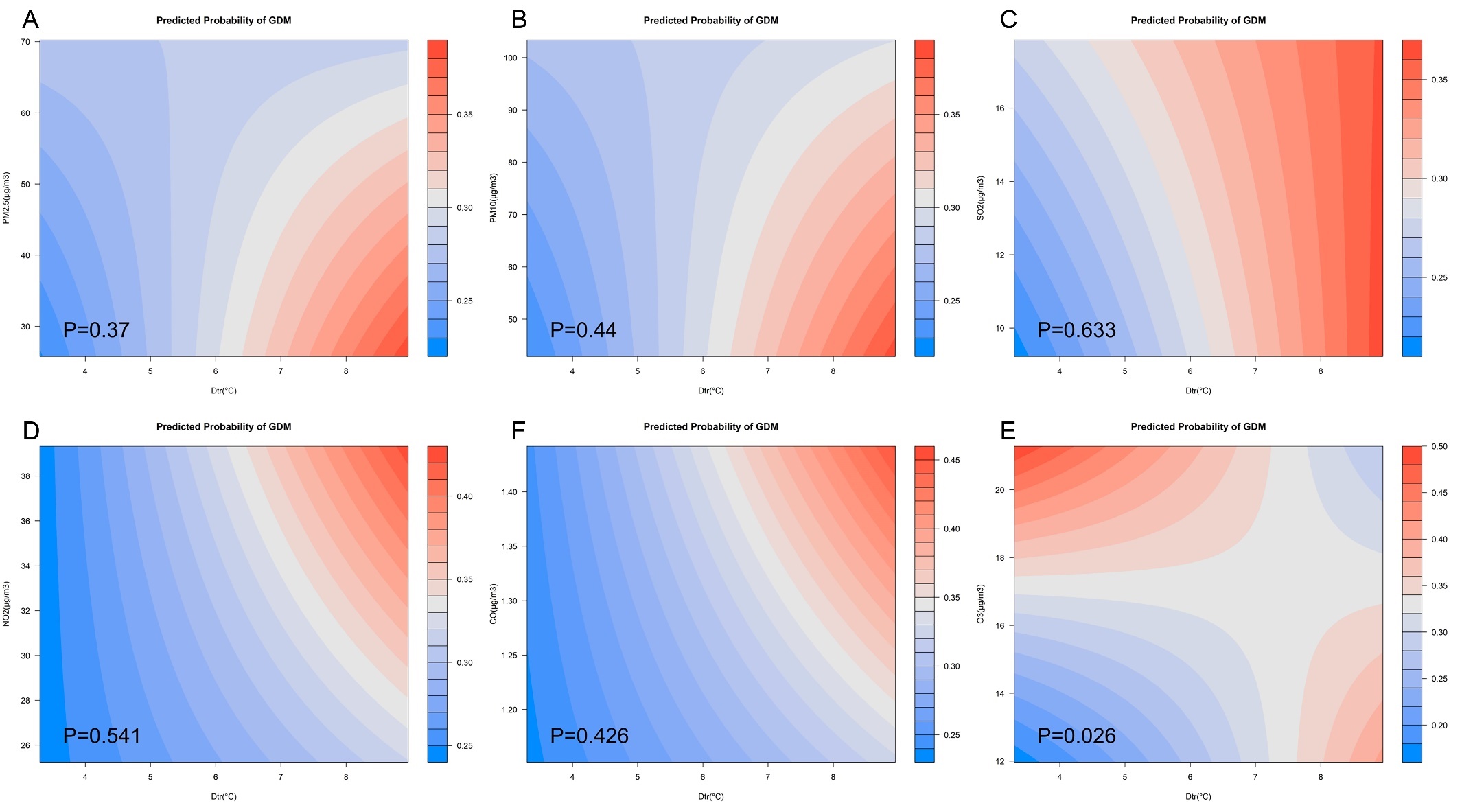


Supplementary Figure 4. Interaction effects of various air pollutants and Dtr during the 2nd trimester on the occurrence of GDM. (A) PM_2.5_ and Dtr; (B) PM_10_ and Dtr; (C) SO_2_ and Dtr; (D) NO_2_ and Dtr; (E) CO and Dtr; (F) O_3_ and Dtr. Interaction effects based on pre-pregnancy BMI, age, in vitro fertilization, scarred uterus, gravidity, and primiparity. The analysis was conducted using a generalized linear model with interaction effects. The color scale on the right side of the contour plot represents the occurrence rate of GDM from the lower to the upper limit.

Abbreviation: DTR: Diurnal Temperature Range; BMI: Body mass index; PM_2.5_: Fine particulate matter; PM_10_: Inhalable particulate matter; SO_2:_ Sulfur dioxide; NO_2_: Nitrogen dioxide; CO: Carbon monoxide; O_3_: Ozone.


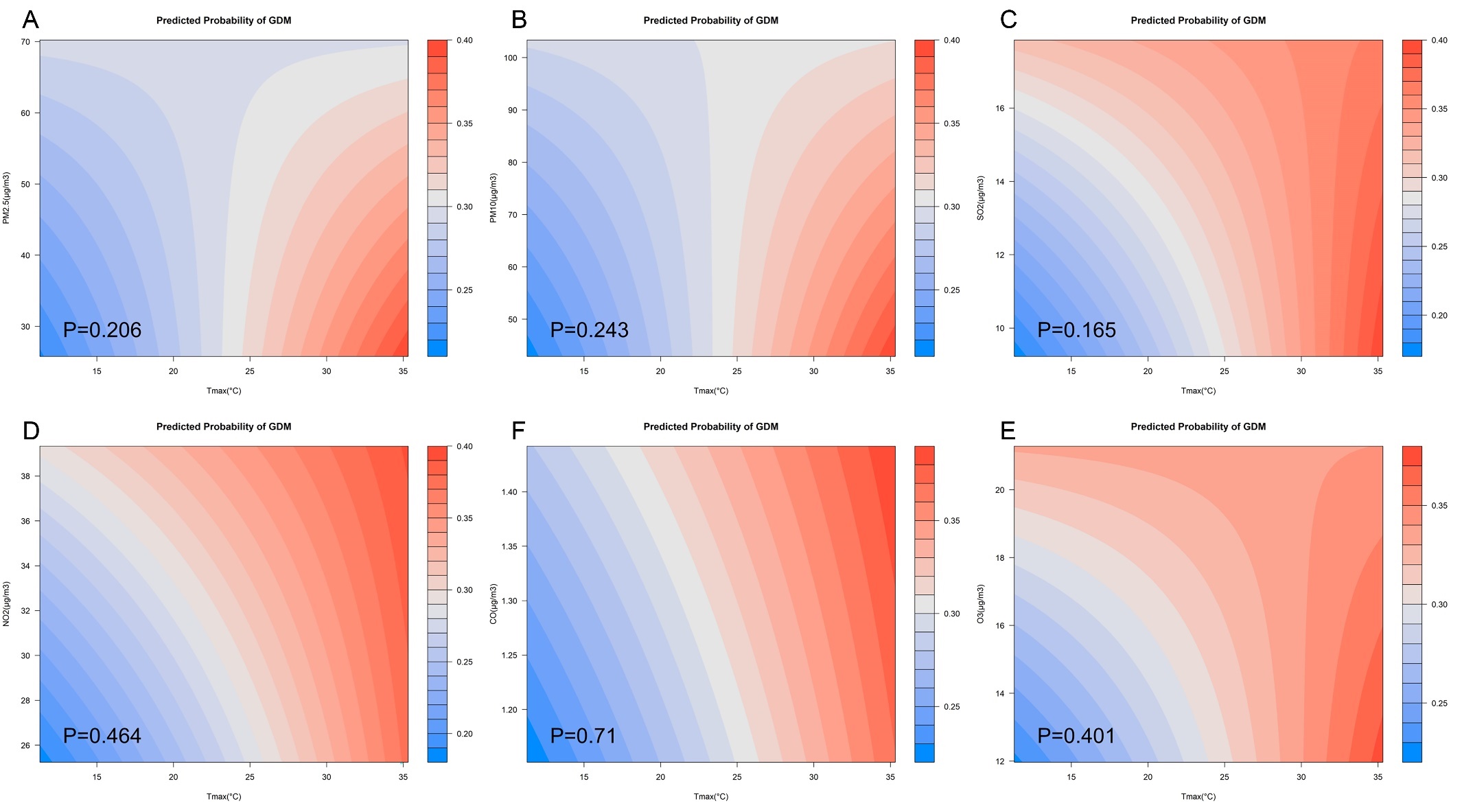


Supplementary Figure 5. Interaction effects of various air pollutants and Tmax during the 2nd trimester on the occurrence of GDM. (A) PM_2.5_ and T_max_; (B) PM_10_ and T_max_; (C) SO_2_ and T_max_; (D) NO_2_ and T_max_; (E) CO and T_max_; (F) O_3_ and T_max_. Interaction effects based on pre-pregnancy BMI, age, in vitro fertilization, scarred uterus, gravidity, and primiparity. The analysis was conducted using a generalized linear model with interaction effects. The color scale on the right side of the contour plot represents the occurrence rate of GDM from the lower to the upper limit.

Abbreviation: T_max_: Maximum Temperature; BMI: Body mass index; PM_2.5_: Fine particulate matter; PM_10_: Inhalable particulate matter; SO_2:_ Sulfur dioxide; NO_2_: Nitrogen dioxide; CO: Carbon monoxide; O_3_: Ozone.


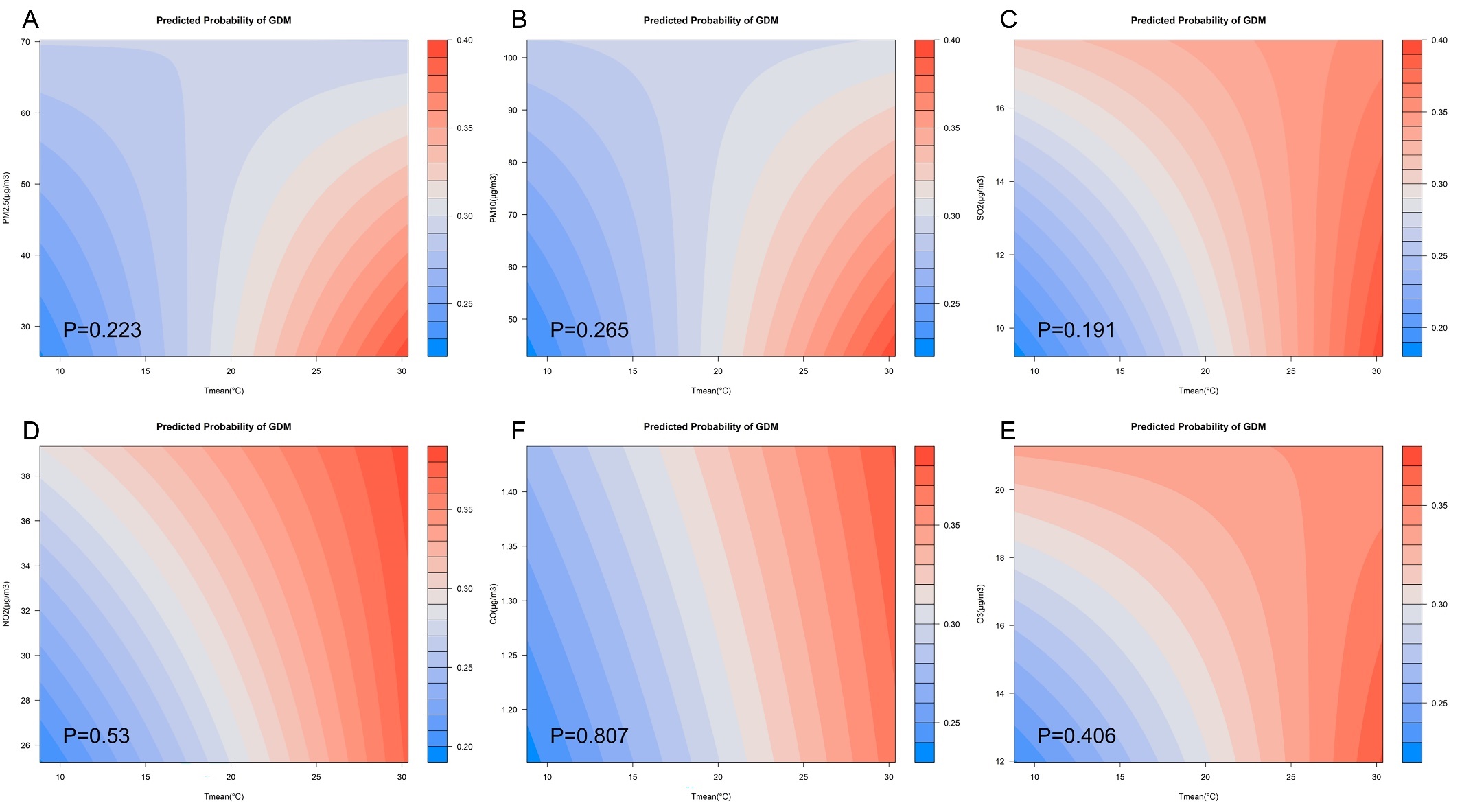


Supplementary Figure 6. Interaction effects of various air pollutants and Tmean during the 2nd trimester on the occurrence of GDM. (A) PM_2.5_ and T_mean_; (B) PM_10_ and T_mean_; (C) SO_2_ and T_mean_; (D) NO_2_ and T_mean_; (E) CO and T_mean_; (F) O_3_ and T_mean_. Interaction effects based on pre-pregnancy BMI, age, in vitro fertilization, scarred uterus, gravidity, and primiparity. The analysis was conducted using a generalized linear model with interaction effects. The color scale on the right side of the contour plot represents the occurrence rate of GDM from the lower to the upper limit.

Abbreviation: T_mean_: Mean Temperature; BMI: Body mass index; PM_2.5_: Fine particulate matter; PM_10_: Inhalable particulate matter; SO_2:_ Sulfur dioxide; NO_2_: Nitrogen dioxide; CO: Carbon monoxide; O_3_: Ozone.


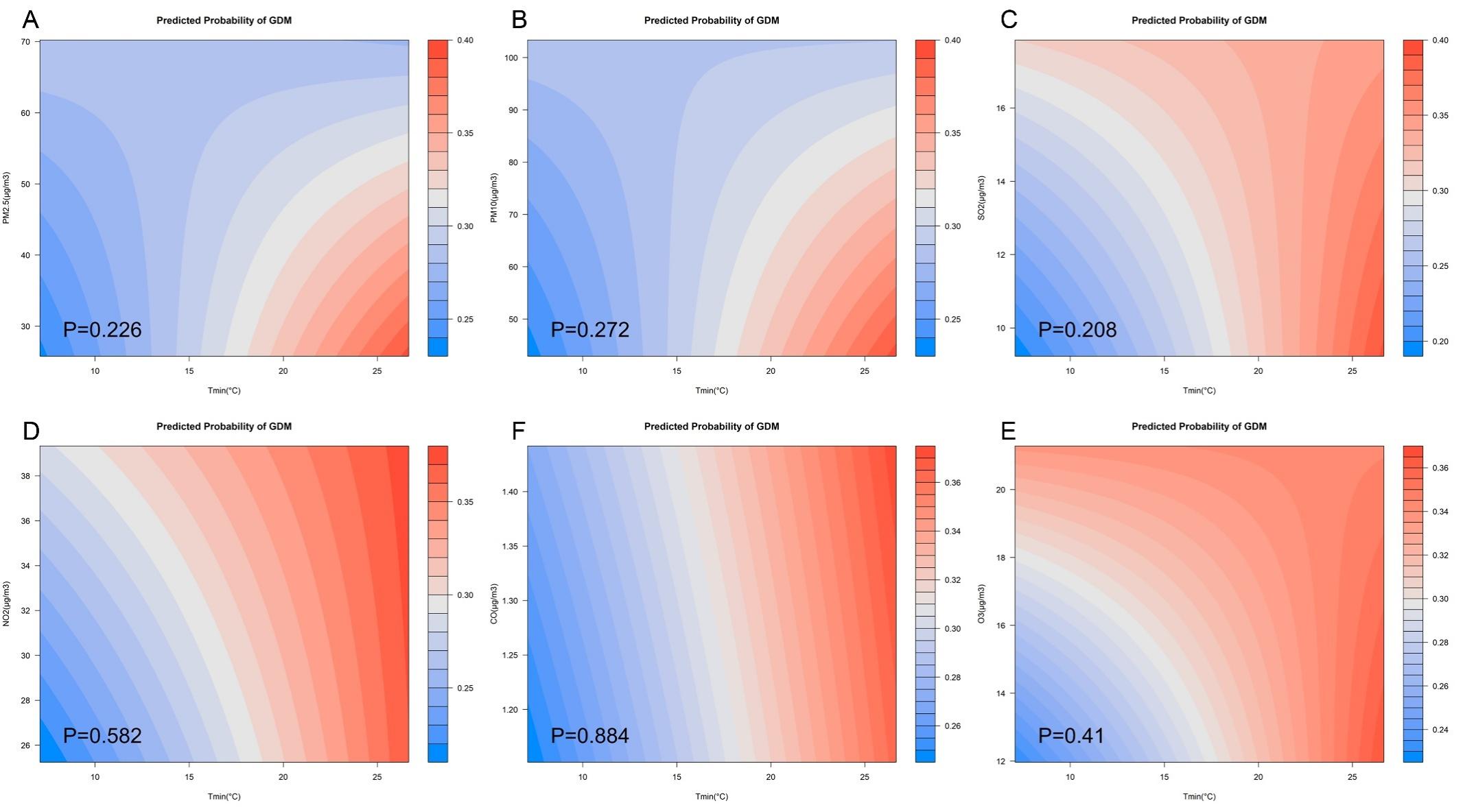


Supplementary Figure 7. Interaction effects of various air pollutants and Tmin during the 2nd trimester on the occurrence of GDM. (A) PM_2.5_ and T_min_; (B) PM_10_ and T_min_; (C) SO_2_ and T_min_; (D) NO_2_ and T_min_; (E) CO and T_min_; (F) O_3_ and T_min_. Interaction effects based on pre-pregnancy BMI, age, in vitro fertilization, scarred uterus, gravidity, and primiparity. The analysis was conducted using a generalized linear model with interaction effects. The color scale on the right side of the contour plot represents the occurrence rate of GDM from the lower to the upper limit.

Abbreviation: T_min_: Minimum Temperature; BMI: Body mass index; PM_2.5_: Fine particulate matter; PM_10_: Inhalable particulate matter; SO_2:_ Sulfur dioxide; NO_2_: Nitrogen dioxide; CO: Carbon monoxide; O_3_: Ozone.

Supplementray Table 1. The average values of environmental temperature and particulate matter during first-trimester to second-trimester.

|  | **T_mean_** | **T_max_** | **T_min_** | **Dtr** | **PM_2.5_** | **PM_10_** | **SO_2_** | **NO_2_** | **CO** | **O_3_** |
| --- | --- | --- | --- | --- | --- | --- | --- | --- | --- | --- |
| First-trimester | 19.33757217 | 23.12547347 | 16.74323428 | 6.382239194 | 43.3590317 | 68.26431477 | 13.10448963 | 32.99047998 | 1.288050733 | 15.27623849 |
| Second-trimester | 19.17045762 | 22.96951762 | 16.58286144 | 6.38665618 | 42.01305765 | 66.39945862 | 12.80836144 | 32.41091802 | 1.274947441 | 15.78601119 |

Abbreviation: T_mean_: Mean Temperature; T_max_: Maximum Temperature; T_min_: Minimum Temperature; DTR: Diurnal Temperature Range; PM_2.5_: Fine particulate matter; PM_10_: Inhalable particulate matter; SO_2:_ Sulfur dioxide; NO_2_: Nitrogen dioxide; CO: Carbon monoxide; O_3_: Ozone.

Supplementray Table 2. Analysis of the correlation between environmental temperature and particulate matter during first-trimester to second-trimester.

|  | aβ (95%CI) | *p-*value | aβ (95%CI) | *p-*value | aβ (95%CI) | *p*-value | aβ (95%CI) | *p-*value |
| --- | --- | --- | --- | --- | --- | --- | --- | --- |
|  | T_mean_ |  | T_max_ |  | T_min_ |  | DTR |  |
| First-trimester |  |  |  |  |  |  |  |  |
| PM_2.5_ | -1.100(-1.132,-1.069) | <0.001* | -0.988(-1.016,-0.960) | <0.001* | -1.221(-1.256,-1.186) | <0.001* | -3.701(-3.836,-3.565) | <0.001* |
| PM_10_ | -1.491(-1.534,-1.447) | <0.001* | -1.337(-1.375,-1.299) | <0.001* | -1.655(-1.704,-1.607) | <0.001* | -4.973(-5.158,-4.789) | <0.001* |
| SO_2_ | -0.196(-0.202,-0.189) | <0.001* | -0.175(-0.181,-0.169) | <0.001* | -0.218(-0.225,-0.211) | <0.001* | -0.633(-0.661,-0.606) | <0.001* |
| NO_2_ | -0.220(-0.230,-0.210) | <0.001* | -0.200(-0.209,-0.192) | <0.001* | -0.242(-0.253,-0.231) | <0.001* | -0.814(-0.851,-0.776) | <0.001* |
| CO | -0.005(-0.005,-0.005) | <0.001* | -0.005(-0.005,-0.004) | <0.001* | -0.005(-0.006,-0.005) | <0.001* | -0.022(-0.023,-0.021) | <0.001* |
| O_3_ | 0.048(0.042,0.054) | <0.001* | 0.046(0.040,0.051) | <0.001* | 0.052(0.046,0.059) | <0.001* | 0.221(0.199,0.244) | <0.001* |
| Second-trimester |  |  |  |  |  |  |  |  |
| PM_2.5_ | -1.224(-1.256,-1.192) | <0.001* | -1.107(-1.135,-1.079) | <0.001* | -1.344(-1.380,-1.308) | <0.001* | -3.96(-4.099,-3.820) | <0.001* |
| PM_10_ | -1.653(-1.696,-1.610) | <0.001* | -1.494(-1.532,-1.456) | <0.001* | -1.817(-1.865,-1.769) | <0.001* | -5.315(-5.503,-5.126) | <0.001* |
| SO_2_ | -0.231(-0.238,-0.225) | <0.001* | -0.209(-0.214,-0.203) | <0.001* | -0.255(-0.262,-0.248) | <0.001* | -0.726(-0.755,-0.698) | <0.001* |
| NO_2_ | -0.292(-0.303,-0.282) | <0.001* | -0.267(-0.276,-0.258) | <0.001* | -0.319(-0.330,-0.307) | <0.001* | -1.022(-1.062,-0.981) | <0.001* |
| CO | -0.006(-0.006,-0.006) | <0.001* | -0.006(-0.006,-0.006) | <0.001* | -0.007(-0.007,-0.006) | <0.001* | -0.026(-0.027,-0.025) | <0.001* |
| O_3_ | 0.124(0.117,0.132) | <0.001* | 0.114(0.107,0.120) | <0.001* | 0.136(0.128,0.144) | <0.001* | 0.428(0.401,0.456) | <0.001* |

Abbreviation: T_mean_: Mean Temperature; T_max_: Maximum Temperature; T_min_: Minimum Temperature; PM_2.5_: Fine particulate matter; PM_10_: Inhalable particulate matter; SO_2_: Sulfur dioxide; NO_2_: Nitrogen dioxide; CO: Carbon monoxide; O_3_: Ozone.

**p* < 0.05

Supplementray Table 3. Spearman analysis results regarding the relationship between six air pollutants.

|  | **PM_2.5_** | **PM_10_** | **SO_2_** | **NO_2_** | **CO** | **O_3_** |
| --- | --- | --- | --- | --- | --- | --- |
|  | **Rho** | **Rho** | **Rho** | **Rho** | **Rho** | **Rho** |
| **First-trimester** |  |  |  |  |  |  |
| PM_2.5_ | 1.000 | 0.997 | 0.971 | 0.936 | 0.872 | -0.699 |
| PM_10_ | 0.997 | 1.000 | 0.979 | 0.937 | 0.870 | -0.684 |
| SO_2_ | 0.971 | 0.979 | 1.000 | 0.967 | 0.884 | -0.710 |
| NO_2_ | 0.936 | 0.937 | 0.967 | 1.000 | 0.919 | -0.830 |
| CO | 0.872 | 0.870 | 0.884 | 0.919 | 1.000 | -0.792 |
| O_3_ | -0.699 | -0.684 | -0.710 | -0.830 | -0.792 | 1.000 |
| **Second-trimester** |  |  |  |  |  |  |
| PM_2.5_ | 1.000 | 0.997 | 0.973 | 0.934 | 0.860 | -0.703 |
| PM_10_ | 0.997 | 1.000 | 0.979 | 0.934 | 0.857 | -0.687 |
| SO_2_ | 0.973 | 0.979 | 1.000 | 0.965 | 0.874 | -0.710 |
| NO_2_ | 0.934 | 0.934 | 0.965 | 1.000 | 0.915 | -0.832 |
| CO | 0.860 | 0.857 | 0.874 | 0.915 | 1.000 | -0.788 |
| O_3_ | -0.703 | -0.687 | -0.710 | -0.832 | -0.788 | 1.000 |

Abbreviation: PM_2.5_: fine particulate matter; PM_10_: inhalable particulate matter; SO_2_: sulfur dioxide; NO_2_: nitrogen dioxide; CO: carbon monoxide; O_3_: ozone

**p* < 0.05

Supplementray Table 4. The influence of the screening season on GDM and its subtypes.

| Season | aOR 95%CI | *p*-value | aOR 95%CI | *p*-value | aOR 95%CI | *p*-value | aOR 95%CI | *p*-value |
| --- | --- | --- | --- | --- | --- | --- | --- | --- |
|  | GDM | | GDM-IFH | | GDM-IPH | | GDM-CH | |
| Winter | 1 (ref.) |  | 1 (ref.) |  | 1 (ref.) |  | 1 (ref.) |  |
| Spring | 1.165(0.951,1.428) | 0.141 | 0.627(0.412,0.944) | 0.027* | 1.395(1.089,1.791) | 0.009* | 1.052(0.737,1.502) | 0.781 |
| Summer | 1.334(1.082,1.644) | 0.007* | 0.417(0.25,0.672) | <0.001* | 2.072(1.623,2.652) | <0.001* | 0.694(0.459,1.038) | 0.079 |
| Autumn | 1.176(0.957,1.447) | 0.124 | 0.716(0.474,1.073) | 0.716 | 1.282(0.994,1.657) | 0.057 | 1.200(0.842,1.712) | 0.313 |

The aOR values were adjusted for age, BMI, whether the woman is primigravida, family history of hyperglycemia, and the season of pregnancy.

Abbreviation: GDM:Gestational diabetes mellitus; GDM-IFH:Gestational diabetes mellitus - impaired fasting hyperglycemia; GDM-IPH:Gestational diabetes mellitus - impaired postprandial hyperglycemia; GDM-CH: Gestational giabetes mellitus - combined hyperglycemia; BMI: Body mass index.

**p* < 0.05

Supplementray Table 5. In the dual-pollutant model, the impact of temperature and air pollutants on OGTT glucose values, GDM, and its subtypes during different periods of pregnancy.

|  | **FBG** |  | **1-h PG** |  | **2-h PG** |  | **AUC for glucose** |  | **GDM** |  | **GDM-IFH** |  | **GDM-IPH** |  | **GDM-CH** |  |
| --- | --- | --- | --- | --- | --- | --- | --- | --- | --- | --- | --- | --- | --- | --- | --- | --- |
| **Variable** | **aβ (95%CI)** | **P-value** | **aβ (95%CI)** | **P-value** | **aβ (95%CI)** | **P-value** | **aβ (95%CI)** | **P-value** | **aOR (95%CI)** | **P-value** | **aOR (95%CI)** | **P-value** | **aOR (95%CI)** | **P-value** | **aOR (95%CI)** | **P-value** |
| **First-trimester** |  |  |  |  |  |  |  |  |  |  |  |  |  |  |  |  |
| **PM2.5 adjusted for CO** | -0.002(-0.005,0.001) | 0.134 | 0.016(0.006,0.025) | 0.001* | 0.019(0.010,0.028) | <0.001* | 0.024(0.011,0.038) | 0.001* | 1.019(1.006,1.032) | 0.005* | 0.988(0.960,1.017) | 0.407 | 1.030(1.015,1.047) | <0.001* | 1.008(0.983,1.033) | 0.542 |
| **PM2.5 adjusted for O3** | -0.002(-0.003,0.000) | 0.097 | 0.005(-0.002,0.012) | 0.139 | 0.007(0.001,0.013) | 0.023* | 0.008(-0.002,0.017) | 0.106 | 1.003(0.994,1.012) | 0.486 | 0.980(0.960,1.000) | 0.045* | 1.010(1.000,1.021) | 0.055 | 0.996(0.980,1.012) | 0.620 |
| **PM10 adjusted for CO** | -0.002(-0.004,-0.000) | 0.043* | 0.009(0.002,0.016) | 0.009* | 0.012(0.006,0.018) | <0.001* | 0.014(0.004,0.024) | 0.004* | 1.011(1.002,1.021) | 0.015* | 0.990(0.970,1.010) | 0.320 | 1.020(1.009,1.031) | <0.001* | 1.002(0.985,1.019) | 0.827 |
| **PM10 adjusted for O3** | -0.001(-0.003,0.000) | 0.051 | 0.003(-0.002,0.007) | 0.274 | 0.004(-0.000,0.008) | 0.054 | 0.004(-0.002,0.010) | 0.228 | 1.002(0.995,1.008) | 0.631 | 0.986(0.972,0.999) | 0.039* | 1.007(0.999,1.014) | 0.072 | 0.996(0.984,1.007) | 0.452 |
| **SO2 adjusted for CO** | -0.015(-0.028,-0.002) | 0.020* | 0.045(-0.002,0.092) | 0.063 | 0.057(0.014,0.101) | 0.010* | -1.860(-4.190,0.4690 | 0.117 | 1.053(0.988,1.122) | 0.110 | 0.937(0.815,1.079) | 0.367 | 1.113(1.032,1.200) | 0.006* | 0.980(0.870,1.104) | 0.738 |
| **SO2 adjusted for O3** | -0.010(-0.018,-0.001) | 0.028* | 0.008(-0.023,0.040) | 0.604 | 0.015(-0.014,0.044) | 0.300 | -0.908(-2.688,0.872) | 0.317 | 0.996(0.954,1.039) | 0.836 | 0.902(0.820,0.992) | 0.034* | 1.030(0.979,1.083) | 0.252 | 0.955(0.884,1.033) | 0.248 |
| **NO2 adjusted for O3** | -0.010(-0.019,-0.002) | 0.012* | 0.007(-0.023,0.036) | 0.661 | 0.010(-0.017,0.037) | 0.475 | 0.006(-0.036,0.048) | 0.767 | 0.989(0.950,1.029) | 0.587 | 0.869(0.793,0.952) | 0.003* | 1.028(0.980,1.078) | 0.258 | 0.955(0.887,1.028) | 0.218 |
| **CO adjusted for PM2.5** | 0.380(-0.050,0.811) | 0.083 | -2.211(-3.772,-0.651) | 0.005* | -2.496(-3.931,-1.061) | 0.001* | -3.269(-5.486,-1.052) | 0.004* | 0.079(0.010,0.657) | 0.019* | 4.555(0.049,422.851) | 0.512 | 0.020(0.002,0.246) | 0.002* | 0.116(0.002,5.772) | 0.280 |
| **CO adjusted for PM10** | 0.478(0.050,0.906) | 0.029* | -1.804(-3.356,-0.251) | 0.023* | -2.147(-3.574,-0.719) | 0.003* | -2.638(-4.844,-0.432) | 0.019* | 0.114(0.014,0.928) | 0.042* | 6.250(0.069,563.790) | 0.425 | 0.025(0.002,0.308) | 0.004* | 0.231(0.005,11.046) | 0.458 |
| **CO adjusted for SO2** | 0.566(0.115,1.018) | 0.014* | -1.384(-3.022,0.255) | 0.098 | -1.520(-3.028,-0.012) | 0.048* | -1.860(-4.190,0.469) | 0.117 | 0.227(0.025,2.056) | 0.187 | 6.119(0.049,759.590) | 0.461 | 0.052(0.004,0.712) | 0.027* | 0.628(0.010,38.083) | 0.824 |
| **CO adjusted for O3** | -0.144(-0.489,0.201) | 0.412 | -0.588(-1.840,0.665) | 0.358 | -0.497(-1.650,0.656) | 0.398 | -0.908(-2.688,0.872) | 0.317 | 0.247(0.045,1.346) | 0.106 | 0.025(0.001,1.052) | 0.053 | 0.402(0.054,3.018) | 0.376 | 0.149(0.007,3.244) | 0.226 |
| **O3 adjusted for PM2.5** | -0.015(-0.028,-0.003) | 0.017* | 0.013(-0.033,0.058) | 0.589 | 0.012(-0.030,0.054) | 0.590 | 0.011(-0.054,0.076) | 0.747 | 0.982(0.923,1.044) | 0.558 | 0.848(0.739,0.974) | 0.020* | 1.015(0.943,1.091) | 0.694 | 1.000(0.895,1.118) | 0.996 |
| **O3 adjusted for PM10** | -0.016(-0.029,-0.004) | 0.009* | 0.006(-0.039,0.050) | 0.806 | 0.005(-0.036,0.046) | 0.807 | -0.000(-0.064,0.064) | 1.000 | 0.977(0.919,1.038) | 0.445 | 0.849(0.741,0.973) | 0.019* | 1.010(0.940,1.084) | 0.791 | 0.991(0.889,1.106) | 0.877 |
| **O3 adjusted for SO2** | -0.018(-0.031,-0.005) | 0.005* | -0.003(-0.049,0.044) | 0.907 | -0.006(-0.049,0.036) | 0.766 | -0.015(-0.081,0.051) | 0.654 | 0.962(0.904,1.025) | 0.230 | 0.838(0.726,0.968) | 0.016* | 0.996(0.925,1.072) | 0.910 | 0.972(0.867,1.090) | 0.632 |
| **O3 adjusted for NO2** | -0.025(-0.041,-0.009) | 0.002* | -0.001(-0.059,0.058) | 0.984 | -0.006(-0.060,0.048) | 0.822 | -0.016(-0.100,0.067) | 0.702 | 0.949(0.877,1.028) | 0.199 | 0.740(0.616,0.889) | 0.001* | 1.010(0.920,1.109) | 0.836 | 0.946(0.818,1.093) | 0.450 |
| **O3 adjusted for CO** | -0.013(-0.028,0.002) | 0.088 | -0.031(-0.085,0.022) | 0.250 | -0.039(-0.089,0.010) | 0.117 | -0.057(-0.133,0.019) | 0.138 | 0.922(0.858,0.991) | 0.028* | 0.825(0.702,0.970) | 0.020* | 0.936(0.859,1.020) | 0.133 | 0.957(0.841,1.090) | 0.511 |
| **Second-trimester** |  |  |  |  |  |  |  |  |  |  |  |  |  |  |  |  |
| **PM2.5 adjusted for CO** | 0.005(0.002,0.007) | <0.001* | 0.002(-0.007,0.011) | 0.692 | -0.002(-0.010,0.007) | 0.665 | 0.003(-0.010,0.017) | 0.622 | 0.999(0.987,1.012) | 0.937 | 1.027(0.999,1.056) | 0.057 | 0.982(0.967,0.997) | 0.020* | 1.023(0.999,1.048) | 0.062 |
| **PM2.5 adjusted for O3** | 0.001(-0.001,0.003) | 0.180 | -0.008(-0.015,-0.002) | 0.015* | -0.010(-0.016,-0.004) | 0.002* | -0.013(-0.022,-0.003) | 0.009* | 0.992(0.983,1.001) | 0.071 | 1.021(1.002,1.041) | 0.028* | 0.976(0.965,0.987) | <0.001* | 1.008(0.992,1.025) | 0.340 |
| **PM10 adjusted for CO** | 0.003(0.001,0.005) | <0.001* | 0.002(-0.005,0.008) | 0.587 | -0.001(-0.007,0.005) | 0.696 | 0.003(-0.006,0.012) | 0.549 | 1.000(0.991,1.009) | 0.980 | 1.020(1.000,1.040) | 0.048* | 0.987(0.977,0.998) | 0.019* | 1.018(1.001,1.035) | 0.039* |
| **PM10 adjusted for O3** | 0.001(-0.000,0.002) | 0.185 | -0.006(-0.010,-0.001) | 0.019* | -0.007(-0.011,-0.003) | 0.002* | -0.008(-0.015,-0.002) | 0.011* | 0.994(0.988,1.001) | 0.078 | 1.015(1.002,1.029) | 0.024* | 0.984(0.976,0.991) | <0.001* | 1.006(0.995,1.018) | 0.289 |
| **SO2 adjusted for CO** | 0.014(0.001,0.027) | 0.031* | -0.010(-0.056,0.035) | 0.656 | -0.019(-0.061,0.023) | 0.374 | -0.013(-0.078,0.052) | 0.695 | 0.963(0.905,1.025) | 0.236 | 1.086(0.946,1.246) | 0.242 | 0.895(0.830,0.965) | 0.004* | 1.068(0.954,1.196) | 0.251 |
| **SO2 adjusted for O3** | 0.001(-0.008,0.010) | 0.839 | -0.050(-0.082,-0.019) | 0.002* | -0.053(-0.082,-0.024) | <0.001* | -0.076(-0.121,-0.031) | 0.001* | 0.944(0.904,0.986) | 0.009* | 1.082(0.987,1.187) | 0.093 | 0.883(0.838,0.930) | <0.001* | 1.017(0.941,1.099) | 0.670 |
| **NO2 adjusted for O3** | -0.001(-0.009,0.008) | 0.851 | -0.053(-0.083,-0.023) | 0.001* | -0.051(-0.079,-0.024) | <0.001* | -0.079(-0.122,-0.036) | <0.001* | 0.943(0.905,0.983) | 0.005* | 1.087(0.996,1.186) | 0.061 | 0.887(0.844,0.932) | <0.001* | 0.996(0.924,1.073) | 0.914 |
| **CO adjusted for PM2.5** | -0.517(-0.949,-0.085) | 0.019* | -2.815(-4.377,-1.254) | <0.001* | -1.972(-3.410,-0.533) | 0.007* | -4.060(-6.279,-1.840) | <0.001* | 0.185(0.022,1.570) | 0.122 | 0.592(0.006,63.738) | 0.826 | 0.455(0.035,5.858) | 0.546 | 0.010(0.000,0.563) | 0.025* |
| **CO adjusted for PM10** | -0.486(-0.913,-0.059) | 0.026* | -2.911(-4.456,-1.366) | <0.001* | -2.002(-3.425,-0.580) | 0.006* | -4.155(-6.351,-1.960) | <0.001* | 0.176(0.021,1.453) | 0.107 | 0.529(0.005,54.304) | 0.788 | 0.444(0.035,5.559) | 0.529 | 0.007(0.000,0.400) | 0.016* |
| **CO adjusted for SO2** | -0.264(-0.718,0.191) | 0.255 | -2.218(-3.859,-0.576) | 0.008* | -1.645(-3.157,-0.133) | 0.033* | -3.172(-5.505,-0.839) | 0.008* | 0.563(0.060,5.317) | 0.616 | 2.436(0.019,304.488) | 0.718 | 1.137(0.073,17.618) | 0.927 | 0.034(0.001,2.031) | 0.105 |
| **CO adjusted for O3** | -0.284(-0.642,0.074) | 0.120 | -2.557(-3.850,-1.264) | <0.001* | -2.448(-3.639,-1.257) | <0.001* | -3.923(-5.761,-2.084) | <0.001* | 0.109(0.018,0.643) | 0.014* | 10.376(0.267,402.858) | 0.210 | 0.016(0.002,0.144) | <0.001* | 0.430(0.017,10.761) | 0.607 |
| **O3 adjusted for PM2.5** | -0.009(-0.022,0.004) | 0.187 | 0.043(-0.004,0.089) | 0.074 | 0.021(-0.022,0.064) | 0.338 | 0.049(-0.018,0.115) | 0.150 | 1.007(0.946,1.072) | 0.824 | 0.966(0.837,1.116) | 0.643 | 0.978(0.907,1.055) | 0.564 | 1.093(0.976,1.224) | 0.125 |
| **O3 adjusted for PM10** | -0.009(-0.022,0.004) | 0.161 | 0.046(-0.000,0.092) | 0.050 | 0.024(-0.019,0.066) | 0.275 | 0.053(-0.012,0.118) | 0.111 | 1.010(0.950,1.074) | 0.750 | 0.966(0.839,1.114) | 0.637 | 0.984(0.914,1.059) | 0.667 | 1.095(0.981,1.223) | 0.106 |
| **O3 adjusted for SO2** | -0.014(-0.027,-0.001) | 0.036* | 0.030(-0.017,0.077) | 0.214 | 0.014(-0.030,0.057) | 0.539 | 0.030(-0.037,0.097) | 0.385 | 0.988(0.927,1.052) | 0.699 | 0.944(0.816,1.093) | 0.442 | 0.964(0.893,1.041) | 0.351 | 1.070(0.955,1.198) | 0.244 |
| **O3 adjusted for NO2** | -0.016(-0.033,0.000) | 0.055 | -0.004(-0.064,0.056) | 0.891 | -0.015(-0.070,0.040) | 0.586 | -0.020(-0.105,0.065) | 0.646 | 0.953(0.879,1.034) | 0.245 | 0.996(0.831,1.193) | 0.962 | 0.902(0.818,0.995) | 0.039* | 1.044(0.903,1.208) | 0.557 |
| **O3 adjusted for CO** | -0.024(-0.039,-0.009) | 0.001* | -0.001(-0.055,0.053) | 0.980 | -0.011(-0.060,0.039) | 0.672 | -0.018(-0.095,0.059) | 0.642 | 0.976(0.908,1.050) | 0.520 | 0.938(0.795,1.107) | 0.450 | 0.963(0.883,1.051) | 0.397 | 1.024(0.899,1.166) | 0.724 |

The aOR values were adjusted for age, BMI, whether the woman is primigravida, family history of hyperglycemia, and the season of pregnancy.

Abbreviation: FBG: Fasting blood glucose; 1h- PG: 1 hour-plasma glucose; 2h- PG: 2 hour-plasma glucose; AUC for glucose: Area under the curve for glucose; GDM: Gestational diabetes mellitus; GDM-IFH: Gestational diabetes mellitus - impaired fasting hyperglycemia; GDM-IPH: Gestational diabetes mellitus - impaired postprandial hyperglycemia; GDM-CH: Gestational giabetes mellitus - combined hyperglycemia; PM_2.5_: Fine particulate matter; PM_10_: Inhalable particulate matter; SO_2_: Sulfur dioxide; NO_2_: Nitrogen dioxide; CO: Carbon monoxide; O_3_: Ozone; BMI: Body mass index.

**p* < 0.05

Supplementray Table 6. The interaction effect between temperature and air pollutants on the incidence of GDM during different periods of pregnancy.

|  | aOR 95%CI | *p*-value | aOR 95%CI | *p*-value | aOR 95%CI | *p*-value | aOR 95%CI | *p*-value |
| --- | --- | --- | --- | --- | --- | --- | --- | --- |
|  | T_mean_ |  | T_max_ |  | T_min_ |  | DTR |  |
| First-trimester |  |  |  |  |  |  |  |  |
| PM_2.5_ | 1.000971(0.999389,1.002554) | 0.229 | 1.000605(0.999178,1.002032) | 0.405 | 1.001337(0.999623,1.003053) | 0.126 | 0.993089(0.986425,0.999773) | 0.043* |
| PM_10_ | 1.000786(0.999639,1.001936) | 0.180 | 1.000511(0.999476,1.001546) | 0.333 | 1.001061(0.999817,1.002310) | 0.095 | 0.995294(0.990528,1.000065) | 0.054 |
| SO_2_ | 1.003747(0.995733,1.011851) | 0.361 | 1.001841(0.994714,1.009035) | 0.614 | 1.005804(0.996999,1.014724) | 0.198 | 0.967696(0.939774,0.996402) | 0.028* |
| NO_2_ | 0.999290(0.993530,1.005117) | 0.810 | 0.998156(0.993034,1.003327) | 0.483 | 1.000588(0.994270,1.006988) | 0.856 | 0.963856(0.945423,0.982659) | <0.001* |
| CO | 0.810368(0.648425,1.012900) | 0.065 | 0.802854(0.659361,0.977585) | 0.029* | 0.823545(0.643416,1.054433) | 0.123 | 0.201250(0.098442,0.410723) | <0.001* |
| O_3_ | 1.001282(0.992134,1.010476) | 0.784 | 1.002475(0.994166,1.010819) | 0.560 | 1.000228(0.990319,1.010191) | 0.964 | 1.051978(1.016440,1.088716) | 0.004* |
| Second-trimester |  |  |  |  |  |  |  |  |
| PM_2.5_ | 0.999159(0.997809,1.000512) | 0.223 | 0.999210(0.997989,1.000434) | 0.206 | 0.999094(0.997630,1.000562) | 0.226 | 0.997199(0.991099,1.003338) | 0.370 |
| PM_10_ | 0.999418(0.998397,1.000441) | 0.265 | 0.999449(0.998524,1.000375) | 0.243 | 0.999378(0.998270,1.000488) | 0.272 | 0.998216(0.993698,1.002751) | 0.440 |
| SO_2_ | 0.995316(0.988347,1.002352) | 0.191 | 0.995512(0.989223,1.001855) | 0.165 | 0.995085(0.987493,1.002757) | 0.208 | 0.993097(0.965245,1.021743) | 0.633 |
| NO_2_ | 0.998531(0.993972,1.003146) | 0.530 | 0.998435(0.994271,1.002647) | 0.464 | 0.998603(0.993664,1.003605) | 0.582 | 1.005839(0.987246,1.024839) | 0.541 |
| CO | 0.977901(0.817603,1.171039) | 0.807 | 0.969384(0.823183,1.142713) | 0.710 | 0.985564(0.811417,1.198719) | 0.884 | 1.333888(0.656759,2.712777) | 0.426 |
| O_3_ | 0.996792(0.989226,1.004332) | 0.406 | 0.996977(0.989911,1.004009) | 0.401 | 0.996588(0.988479,1.004678) | 0.410 | 0.959711(0.925400,0.994970) | 0.026* |

The aOR values were adjusted for age, BMI, whether the woman is primigravida, family history of hyperglycemia, and the season of pregnancy.

Abbreviation: T_mean_: Mean temperature; T_max_: Maximum temperature; T_min_: Minimum temperature; DTR: Diurnal temperature range; PM_2.5_: Fine particulate matter; PM_10_: Inhalable particulate matter; SO_2_: Sulfur dioxide; NO_2_: Nitrogen dioxide; CO: Carbon monoxide; O_3_: Ozone; BMI: Body mass index.

**p* < 0.05
